# Recombinant RSV G protein vaccine induces enhanced respiratory disease via IL-13 and mucin overproduction

**DOI:** 10.1038/s41541-024-00987-w

**Published:** 2024-10-12

**Authors:** Eigo Kawahara, Kota Senpuku, Yoshino Kawaguchi, Shinya Yamamoto, Koubun Yasuda, Etsushi Kuroda, Noriko Ouji-Sageshima, Toshihiro Ito, Toshiro Hirai, Takehiko Shibata, Yasuo Yoshioka

**Affiliations:** 1 Laboratory of Nano-Design for Innovative Drug Development, Graduate School of Pharmaceutical Sciences, Osaka University, Osaka, Japan; 2 Vaccine Creation Group, Research Institute for Microbial Diseases, Osaka University, Osaka, Japan; 3 Department of Pharmacokinetics and Biopharmaceutics, Institute of Biomedical Sciences, Tokushima University, Tokushima, Japan; 4 The Research Foundation for Microbial Diseases of Osaka University, Osaka, Japan; 5 Institute for Open and Transdisciplinary Research Initiatives, Osaka University, Osaka, Japan; 6 Department of Immunology, Hyogo College of Medicine, Hyogo, Japan; 7 Department of Immunology, Nara Medical University, Nara, Japan; 8 Center for Advanced Modalities and DDS, Osaka University, Osaka, Japan; 9 Department of Microbiology, Tokyo Medical University, Tokyo, Japan; 10 Global Center for Medical Engineering and Informatics, Osaka University, Osaka, Japan; 11 Center for Infectious Disease Education and Research, Osaka University, Osaka, Japan

**Keywords:** Protein vaccines, Viral host response

## Abstract

The G protein expressed on the surface of respiratory syncytial virus (RSV) is important for adhesion to host cells and as a vaccine target antigen. The corresponding vaccines can effectively eliminate RSV. However, they exacerbate pulmonary immunopathology including eosinophilic infiltration in the lungs after an RSV challenge in animal models, raising concerns about enhanced respiratory disease (ERD); thus, approaches that mitigate these effects are urgently needed. Herein, we aimed to examine the mechanisms of G protein vaccine-induced ERD in mice, using recombinant G protein as a vaccine antigen. After the RSV challenge, G protein-vaccinated mice exhibited lung weight gain, lung tissue damage, and increased infiltration of eosinophils, neutrophils, and CD4^+^ T cells into the lungs. We set lung weight gain as the endpoint for ERD and examined the impact of each infiltrating cell on lung weight gain. We observed that CD4^+^ T cells, but not eosinophils or neutrophils, that infiltrate the lungs are responsible for lung weight gain. In addition, T helper 2 cell-mediated IL-13 induced mucin hypersecretion and lung weight gain. Mucin hypersecretion may contribute to weight gain in the lungs. In conclusion, our results indicate a novel mechanism of G protein vaccine-induced ERD via IL-13 and mucin hypersecretion, which could lead to the development of safe G protein vaccines and the elucidation of the causes of ERD associated with other vaccines.

## Introduction

Respiratory syncytial virus (RSV) affects nearly every infant before the age of 2 years, and subsequent reinfections are common^1,2^. RSV commonly targets the upper respiratory tract, resulting in symptoms resembling a cold, such as runny nose and cough^2,3^. However, in infants younger than 6 months and older adults with underlying medical conditions, RSV has a high rate of lower respiratory tract spread, causing diseases such as pneumonia and bronchiolitis^2,3^. The RSV infects 64 million people annually and causes 160,000 deaths worldwide each year^4,5^, creating a need for vaccines.

The G and F proteins are expressed on the surface of RSV^1,2^. RSV is transmitted by adhesion to host cells via the G protein, followed by membrane fusion with host cells via the F protein^1,2^, making them important for infection and as vaccine target antigens. A vaccine targeting the F protein was approved in 2023 as the world’s first RSV vaccine. Clinical trials have shown that recombinant F protein vaccines are highly effective against severe RSV-related lower respiratory tract diseases, with an efficacy rate of over 90% in older adults^6^. In addition, infants whose mothers received the vaccine during pregnancy exhibited approximately 70% efficacy within 180 days of birth^7^. However, its efficacy against RSV-related acute respiratory infections is lower, at approximately 60–70% in older adults and less than 40% in infants within 180 days of birth^6–8^, suggesting room for improvement.

Vaccines targeting the G protein have not been approved but have been reported to be effective in eliminating RSV in both animal models^9,10^. In addition, G protein vaccines are expected to be effective in combination with F protein vaccines because the inhibitory phase of infection differs between the vaccine types^10–12^. Vaccines containing recombinant G proteins produced using mammalian cells or G protein-expressing vaccinia viral vectors are effective in eliminating RSV. However, they can induce pulmonary immunopathology, such as excessive eosinophil infiltration, after an RSV challenge in animal models, which is not observed in unvaccinated controls^12–15^. Thus, G protein vaccines induce enhanced respiratory disease (ERD), raising safety concerns^10^. ERD is a type of adverse reaction that refers to the worsening of symptoms following viral infection due to vaccination. As ERD has been previously reported in human clinical trials of the formalin-inactivated RSV (FI-RSV) vaccine, it is of particular concern in case of RSV vaccines^16,17^. Some FI-RSV vaccine recipients developed severe lower respiratory disease with excessive eosinophil infiltration after an RSV infection and died, whereas unvaccinated patients rarely experienced severe disease after the infection^16,17^. In recent years, we and other groups have reported the optimized recombinant G protein vaccines with reduced lung pathogenesis following RSV challenge in animal models^12,15,18^, one of which has progressed to clinical trials^19^. However, G protein vaccine-induced ERD has not been elucidated completely, and more information is needed to advance the clinical use of G protein vaccines.

In this study, to enhance the safety of G protein vaccines for clinical use, we aimed to examine the mechanisms of G protein vaccine-induced ERD in mice, using recombinant G protein produced in mammalian cells as a vaccine antigen.

## Results

### G protein vaccine promotes viral clearance but induces pulmonary inflammatory responses

**Fig. 1:**
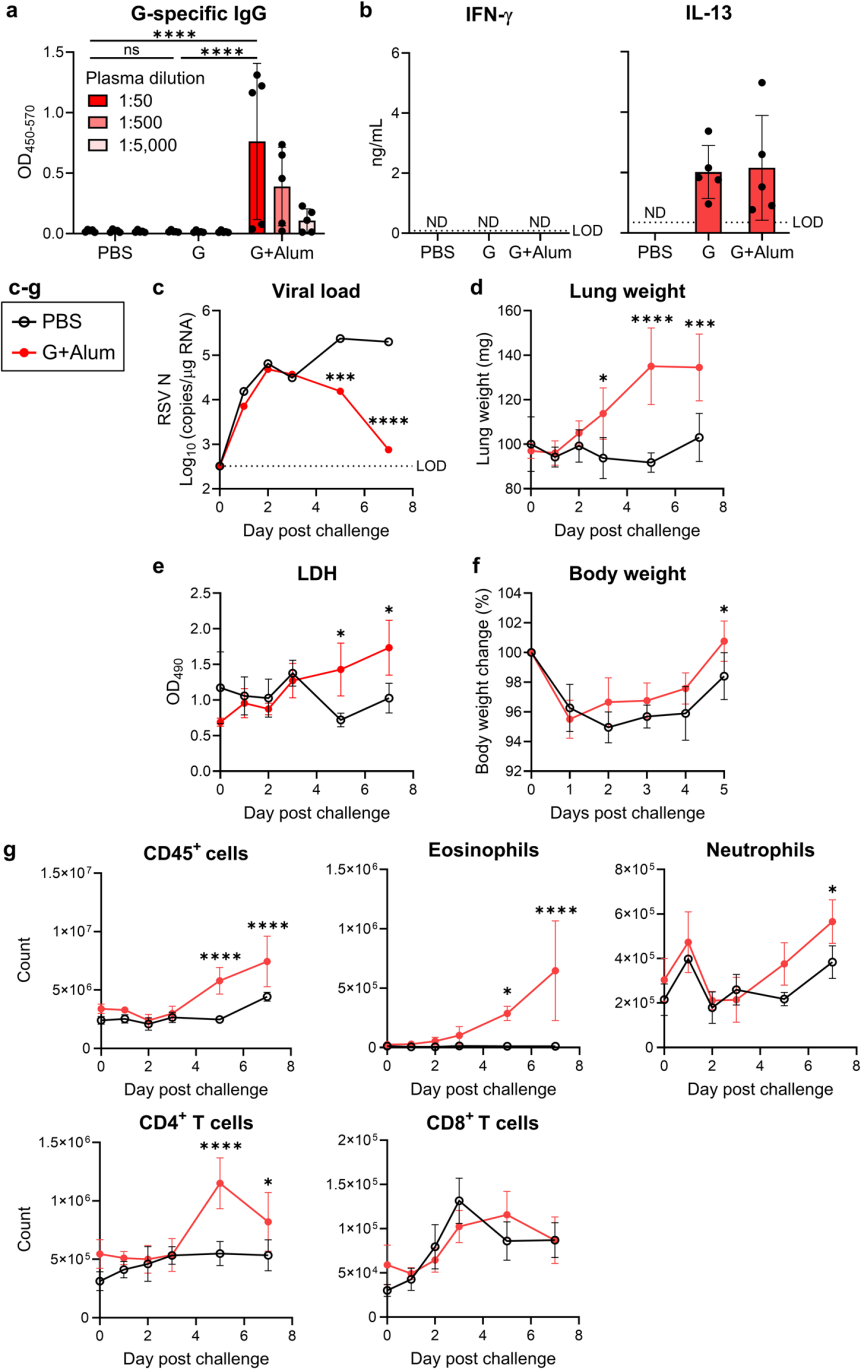
G protein vaccine-induced immune responses and pulmonary inflammatory responses. **a**–**g** Mice were vaccinated with the G protein or G protein plus aluminum hydroxide (Alum). **a** Plasma levels of G-specific total IgG. **b** Cytokine levels in the supernatant after restimulation of splenocytes with G protein for 3 days. **c–g** G+Alum-vaccinated mice were challenged with the RSV. **c** The viral loads in the right lung samples as determined by the mRNA levels of RSV nucleoprotein (RSV N). **d** Right lung weight. **e** Levels of lactate dehydrogenase (LDH) in bronchoalveolar lavage fluid (BALF). **f** Body weight change. **g** Number of CD45^+^ cells, eosinophils, neutrophils, CD4^+^ T cells, and CD8^+^ T cells in the left lung. **a**–**g** Each experiment was performed (**a**) three times or (**b**–**g**) twice. **a**, **b**, **f** *n* = 5 per group. **c**, **d**, **g** *n* = 4 per group. **e** *n* = 3 per group. **a**–**g** Data are presented as (**a**, **b**, **d**–**g**) the mean ± SD or (**c**) median. **a**–**g** *P < 0.05, ***P < 0.001, ****P < 0.0001 as indicated using (**a**) two-way ANOVA with Tukey’s test, (**b**) one-way ANOVA with Tukey’s test, or (**c**–**g**) two-way ANOVA with Sidak’s test. ND not detected. ns not statistically significant. LOD limit of detection.

First, to confirm the immunogenicity of the G protein produced using mammalian cells, we vaccinated mice subcutaneously with G-alone or G plus aluminum hydroxide (Alum) and analyzed the antibody and T-cell responses. The G-alone vaccine did not induce the production of detectable G-specific IgG in the plasma, similar to the PBS vaccine. Meanwhile, the G+Alum vaccine significantly increased the levels of G-specific IgG compared to those observed in the PBS- or G-alone-vaccinated groups (Fig. 1a). When restimulated with the G protein, splenocytes from mice vaccinated with G-alone or G+Alum produced a T helper 2 (Th2) cytokine (interleukin [IL]-13), but not a T helper 1 (Th1) cytokine (interferon [IFN]-γ), compared to those from PBS-vaccinated mice (Fig. 1b). Thus, the G-alone vaccine induced G-specific Th2 responses but not G-specific IgG, whereas the G+Alum vaccine induced both G-specific Th2 responses and G-specific IgG.

To investigate G protein vaccine-induced ERD, we analyzed the lung samples obtained from day 0 to 7 after the RSV challenge in the G+Alum-vaccinated mice. First, we measured the viral load in the lungs by real-time reverse transcription polymerase chain reaction (RT-PCR) (Fig. 1c). In PBS-vaccinated mice, the viral load increased from post challenge day 0 to day 5, whereas in the G+Alum-vaccinated mice, the viral load peaked on post challenge day 2 and decreased thereafter until day 7 (Fig. 1c).

Next, we evaluated the lung inflammatory responses. The G+Alum-vaccinated mice showed a significant increase in lung weight from day 3 to day 7 after the RSV challenge, in contrast to the PBS-vaccinated mice, which showed no change in lung weight over time (Fig. 1d). The G+Alum vaccinated mice showed increased levels of lactate dehydrogenase (LDH), a marker of tissue damage, in the bronchoalveolar lavage fluid (BALF) on post challenge days 5 and 7, compared to those in the PBS-vaccinated group (Fig. 1e). However, the body weight of G+Alum mice was not lower than that of the PBS vaccinated mice (Fig. 1f).

We analyzed the infiltrating cells in the lungs using flow cytometry (Fig. 1g, Supplementary Fig. 1). The G+Alum vaccine significantly increased the number of CD45^+^ leukocytes on post challenge days 5 and 7, compared to those in the PBS-vaccinated group (Fig. 1g). Among leukocytes, the G+Alum vaccine increased the number of eosinophils on post challenge days 5 and 7, compared to those in the PBS-vaccinated group (Fig. 1g). In addition, the G+Alum vaccine significantly increased the number of neutrophils on post challenge day 7 compared to that in the PBS-vaccinated group (Fig. 1g). Furthermore, the G+Alum vaccine significantly increased the number of CD4^+^ T cells on post challenge days 5 and 7 compared to those in the PBS-vaccinated group, whereas there was no difference in the number of CD8^+^ T cells (Fig. 1g). These results indicate that the G+Alum vaccine promotes viral clearance but induces pulmonary inflammatory responses, such as lung weight gain, tissue damage, and infiltration of eosinophils, neutrophils, and CD4^+^ T cells into the lung.

### Eosinophils and neutrophils do not increase lung weight while involve in viral clearance

**Fig. 2:**
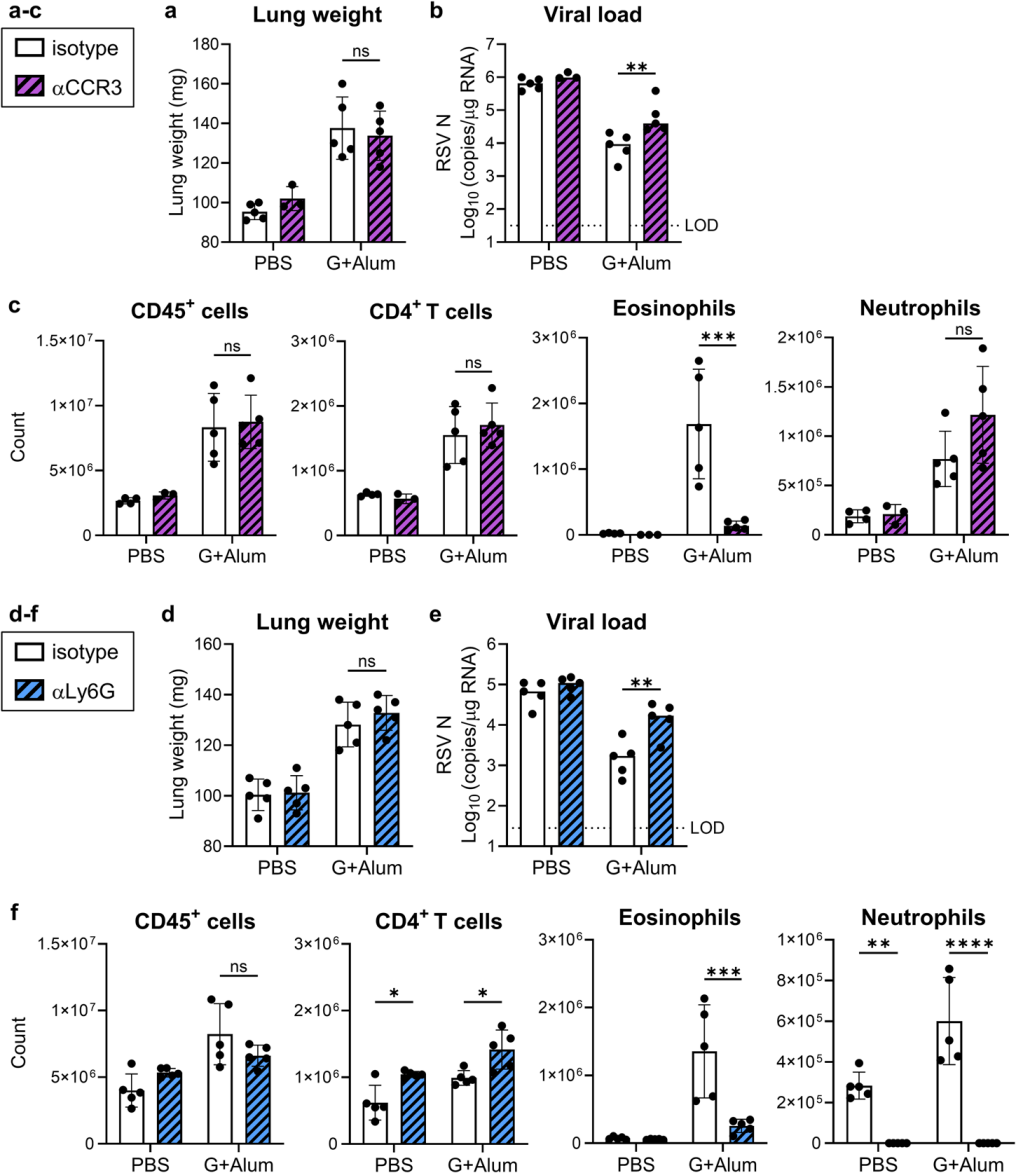
Effect of eosinophils and neutrophils on G+Alum vaccine-induced lung weight gain. **a**–**f** G+Alum-vaccinated mice were challenged with RSV and analyzed on day 5 post challenge. **a**–**c** Before and after challenge, mice were treated with anti-CCR3 antibody (αCCR3) or IgG2b isotype control (Ctrl-IgG). **a** Right lung weight. **b** The viral loads in the right lungs. **c** Number of CD45^+^ cells, CD4^+^ T cells, eosinophils, and neutrophils in the left lung. **d**–**f** Before and after the challenge, the mice were treated with anti-Ly6G antibody (αLy6G) or IgG2a isotype control (Ctrl-IgG). **d** Right lung weight. **e** Viral loads in the right lungs. **f** Number of CD45^+^ cells, CD4^+^ T cells, eosinophils, and neutrophils in the left lung. **a**–**f** Each experiment was performed twice. **a**–**c** *n* = 3–5 per group. **d**–**f** *n* = 5 per group. **a**–**f** Data are presented as (**a**, **c**, **d**, **f**) the mean ± SD or (**b**, **e**) median. **a**–**f** *P < 0.05, **P < 0.01, ***P < 0.001, ****P < 0.0001 as indicated using two^-^way ANOVA with Tukey’s test. ns not statistically significant. LOD limit of detection.

Using lung weight gain as the endpoint for pulmonary inflammatory responses, we analyzed the effect of each infiltrating cell on lung weight gain on day 5 after challenge in the G+Alum-vaccinated mice when the lung weight was maximal. First, to analyze the effect of eosinophils, we depleted eosinophils by administering an anti-CCR3 antibody before the RSV challenge (Supplementary Fig. 2a). In the G+Alum-vaccinated mice, the eosinophil depletion did not alter the lung weight compared to that in the control antibody-treated group (Fig. 2a). Meanwhile, the eosinophil depletion significantly increased the viral load compared to that in the control antibody-treated group in G+Alum-vaccinated mice (Fig. 2b). In addition, the eosinophil depletion did not change the counts of CD45^+^ cells, neutrophils, or CD4^+^ T cells compared to the control antibody-treated group but decreased the number of eosinophils (Fig. 2c). This evidence suggests that eosinophils were not involved in lung weight gain but were involved in viral clearance in G+Alum-vaccinated mice.

Next, we depleted neutrophils using anti-Ly6G antibody administration before the RSV challenge and analyzed the effect of neutrophils on lung weight gain (Supplementary Fig. 2b). Approximately 70% of the eosinophils infiltrating the lungs after the RSV challenge in G+Alum-vaccinated mice expressed low to moderate levels of Ly6G (Supplementary Fig. 2b). These eosinophils were depleted alongside neutrophils after treatment with an anti-Ly6G antibody (Supplementary Fig. 2b). In the G+Alum-vaccinated mice, the neutrophil depletion did not alter lung weight compared to that in the control antibody-treated group (Fig. 2d). Meanwhile, the neutrophil depletion significantly increased the viral load compared with that in the control antibody-treated group in the G+Alum-vaccinated mice (Fig. 2e). In addition, the neutrophil depletion significantly reduced the number of eosinophils and neutrophils in the G+Alum-vaccinated mice, whereas the number of CD45^+^ cells remained unchanged compared to that in the control antibody-treated group (Fig. 2f). The neutrophil depletion slightly but significantly increased the number of CD4^+^ T cells compared to that in the control antibody group in both PBS- and G+Alum-vaccinated mice (Fig. 2f). Thus, neutrophils did not affect lung weight gain.

### CD4^+^ T cells promote lung weight gain after RSV challenge

**Fig. 3:**
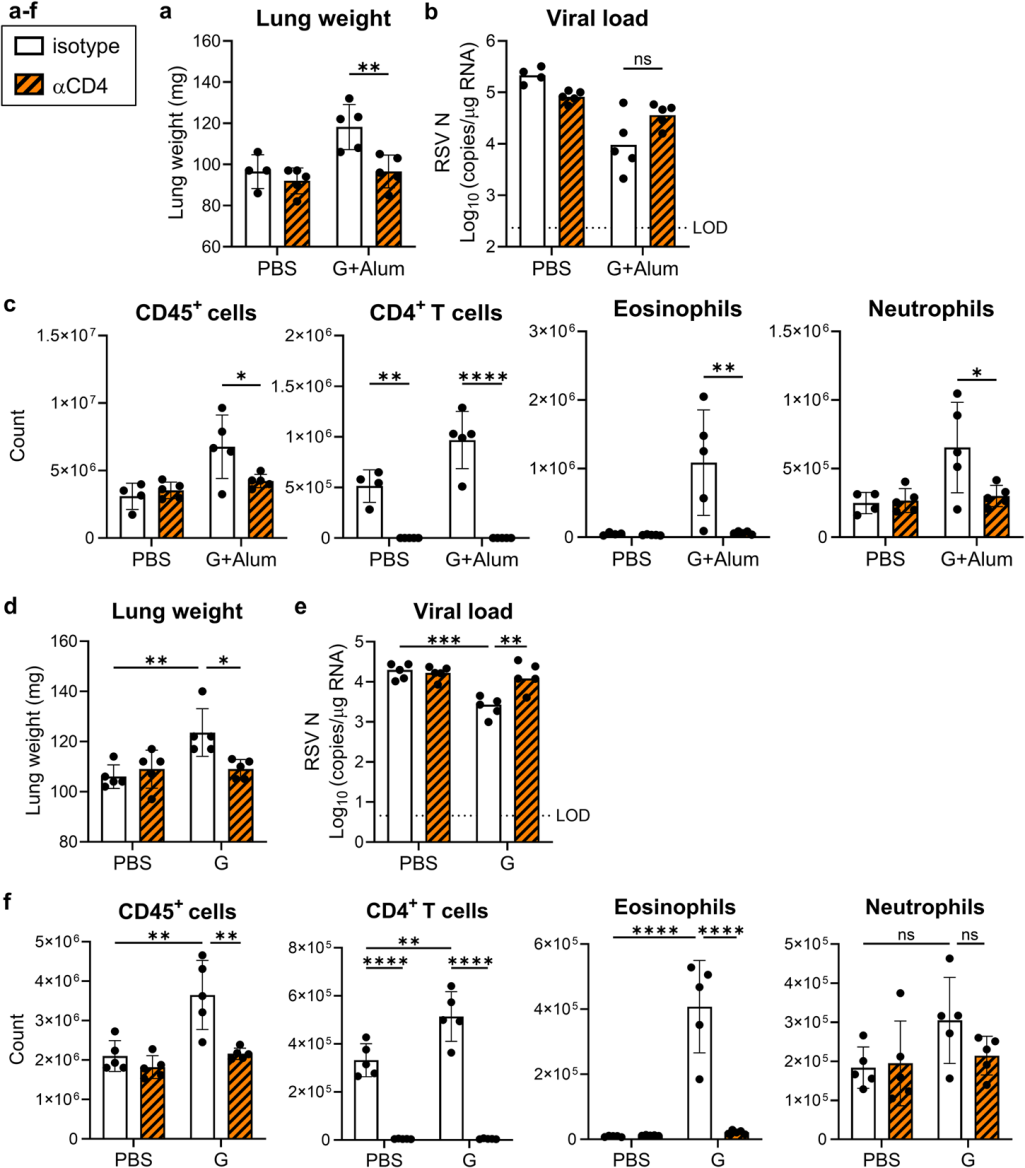
Effect of CD4^+^ T cells on G protein vaccine-induced lung weight gain. **a**–**c** G+Alum-vaccinated mice were challenged with the RSV and analyzed on day 5 post challenge. Before the RSV challenge, the mice were treated with anti-CD4 antibody (αCD4) or IgG2b isotype control (Ctrl-IgG). **a** Right lung weight. **b** Viral loads in the right lungs. **c** Number of CD45^+^ cells, CD4^+^ T cells, eosinophils, and neutrophils in the left lung. **d**–**f** G-alone-vaccinated mice were challenged with the RSV and analyzed on day 5 post challenge. Before the RSV challenge, the mice were treated with αCD4 or Ctrl-IgG. **d** Right lung weight. **e** Viral loads in the right lung samples. **f** Number of CD45^+^ cells, CD4^+^ T cells, eosinophils, and neutrophils in the left lung. **a**–**f** Each experiment was performed twice. **a**–**c** *n* = 4–5 per group. **d**–**f** *n* = 5 per group. **a**–**f** Data are presented (**a**, **c**, **d**, **f**) as the mean ± SD or (**b**, **e**) median. **a**–**f** *P < 0.05, **P < 0.01, ***P < 0.001, ****P < 0.0001 as indicated using two^-^way ANOVA with Tukey’s test. ns not statistically significant. LOD limit of detection.

We depleted CD4^+^ T cells using anti-CD4 antibody administration before the RSV challenge and analyzed the effect of CD4^+^ T cells on lung weight gain (Supplementary Fig. 2c, d). In the G+Alum-vaccinated mice, the CD4^+^ T cell depletion significantly reduced lung weight to the same level as that in PBS-vaccinated mice (Fig. 3a). Meanwhile, the CD4^+^ T cell depletion tended to increase the viral load compared to that in the control antibody-treated group in G+Alum-vaccinated mice (Fig. 3b). In addition, the CD4^+^ T cell depletion significantly reduced the number of CD45^+^ cells, eosinophils, neutrophils, and CD4^+^ T cells compared to those in the control antibody-treated group in G+Alum-vaccinated mice (Fig. 3c). Furthermore, we examined whether the CD4^+^ T-cell-dependent lung weight gain observed in the G+Alum-vaccinated mice was also observed in the G-alone-vaccinated mice. The G-alone vaccine significantly increased the lung weight but decreased the viral load when compared to that in the PBS-vaccinated mice (Fig. 3d, e). In addition, the G-alone vaccine significantly increased the number of CD45^+^ cells, CD4^+^ T cells, and eosinophils when compared to that in the PBS-vaccinated mice (Fig. 3f). In the G-alone-vaccinated mice, CD4^+^ T cell depletion significantly reduced the lung weight and number of CD45^+^ cells, eosinophils, and CD4^+^ T cells when compared to that in the control antibody-treated group, while significantly increasing the lung viral load (Fig. 3d–f). Thus, CD4^+^ T cells were involved in lung weight gain in the G+Alum- and G-alone-vaccinated mice.

### CD4^+^ T cells are the primary drivers of ERD

**Fig. 4:**
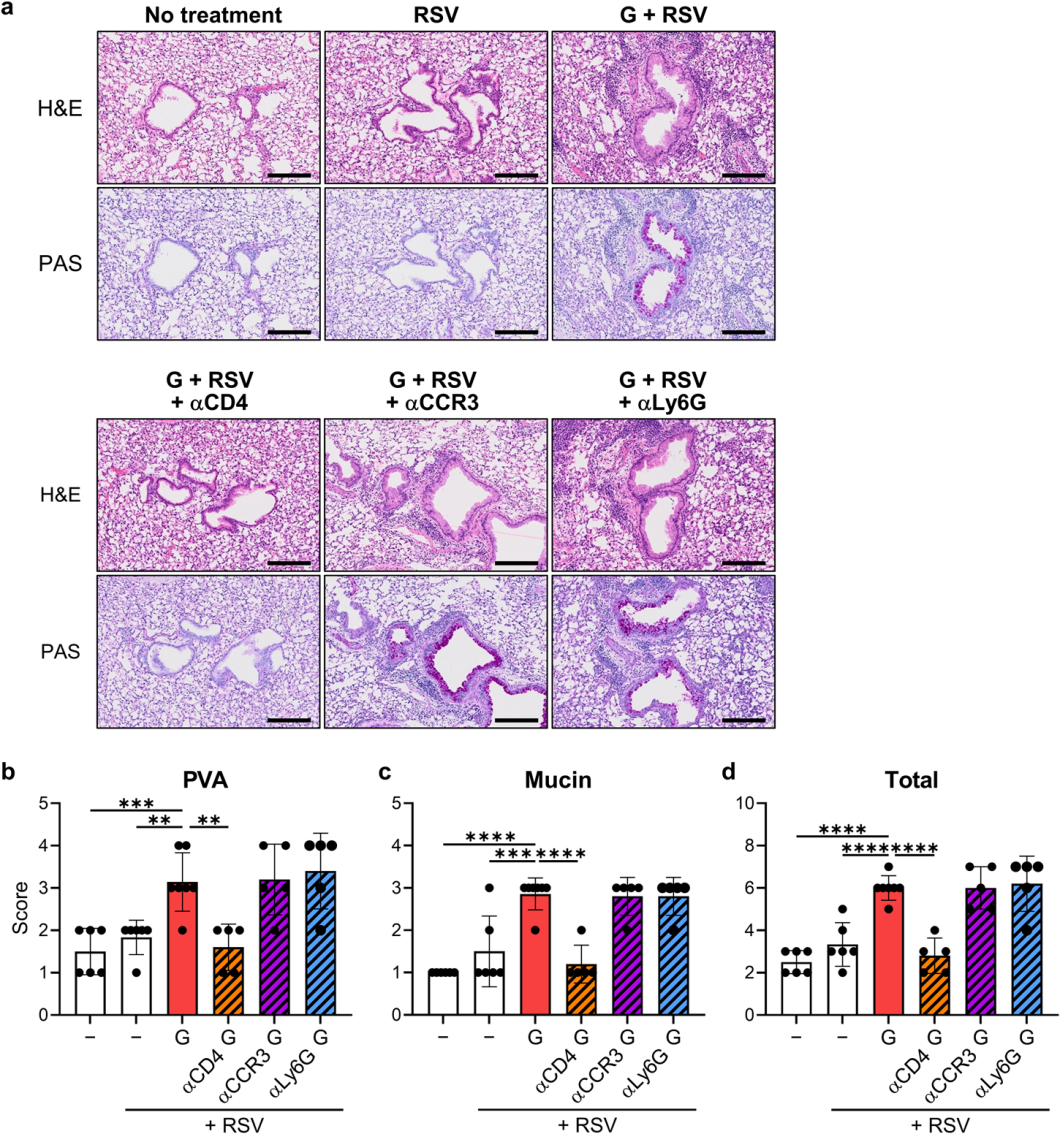
Histopathological analysis of lungs following the RSV challenge in G protein-vaccinated mice. **a**–**d** Mice vaccinated by the G-alone vaccine were challenged with the RSV and analyzed on day 5 post-challenge. Before or after the RSV challenge, the mice were treated with an anti-CD4 antibody (αCD4), anti-CCR3 antibody (αCCR3), or anti-Ly6G antibody (αLy6G). **a** Representative photomicrographs of lung sections stained with hematoxylin and eosin (H&E) or periodic acid-Schiff (PAS). Scale bar, 200 μm. **b**–**d** Semi-quantitative histology scores for (**b**) perivascular aggregates of leukocytes (PVA), (**c**) mucin, and (**d**) total histology (PVA + mucin). **a**–**d** Each experiment was performed twice. **b**–**d** *n* = 5–7 per group. Data are presented as the mean ± SD. **P < 0.01, ***P < 0.001, ****P < 0.0001 as indicated using one-way ANOVA with Dunnett’s test compared with G + RSV.

We have shown above the effect of each immune cell on lung weight gain. To examine the findings via histopathology, we analyzed the lungs using hematoxylin and eosin (H&E) staining and periodic acid-Schiff (PAS) staining. Compared with the no treatment group, RSV infection induced a lower cellular infiltration and detectable mucin secretion in the bronchioles (Fig. 4a). RSV challenge after G-alone vaccination induced severe cellular infiltration in bronchioles, alveoli, and interstitium, and mucin hypersecretion and goblet cell hyperplasia in bronchioles compared with no treatment or RSV infection alone (Fig. 4a). In mice vaccinated with the G-alone vaccine, CD4^+^ T cell depletion markedly reduced infiltration of cells into the bronchioles, alveoli, and interstitium, and mucin secretion and goblet cell hyperplasia in the bronchioles (Fig. 4a). However, depletion of eosinophils or neutrophils did not alter cellular infiltration into the bronchioles, alveoli, and interstitium, nor mucin hypersecretion and goblet cell hyperplasia in the bronchioles (Fig. 4a). In addition, RSV challenge after G-alone vaccination significantly increased the histology scores for perivascular aggregates of leukocytes (PVA) and mucin compared with no treatment or RSV infection alone, consequently significantly increasing the total histology score (Fig. 4b–d). In mice vaccinated with the G-alone vaccine, CD4^+^ T cell depletion, but not eosinophil or neutrophil depletion, significantly reduced the PVA, mucin, and total histology scores (Fig. 4b–d). Thus, pathologically, CD4^+^ T cells are involved in the pulmonary pathogenesis of ERD, which correlates with lung weight gain.

To further comprehensively assess the ERD induced by CD4^+^ T cells, we conducted RNA sequencing (RNA-seq) of lung samples after RSV challenge in G-alone-vaccinated mice with or without anti-CD4 antibody treatment. Principal component analysis and heatmap revealed distinctly different gene expression patterns in mice vaccinated with the G-alone vaccine compared to PBS-vaccinated mice or CD4^+^ T cell-depleted G-alone-vaccinated mice (Supplementary Fig. 3a, b). The mice vaccinated with the G-alone vaccine showed upregulation of 314 genes compared with the PBS-vaccinated mice, and most of this upregulated gene expression was abolished by CD4^+^ T cell depletion (Supplementary Fig. 3c). Specifically, the G-alone vaccine significantly increased the expression of *Il13*, IL-13-related genes (*Muc5ac, Chil3, Fxyd4, Slc5a1*, and *Sprr2a*)^20–22^, eosinophil-related gene *Rnase2a*^23,24^, and other Th2 response-related gene *Ccl8*^25,26^ (compared to the PBS vaccine or G-alone vaccine plus anti-CD4 antibody treatment) (Supplementary Fig. 3c). In addition, the differentially expressed genes (DEGs) in mice vaccinated with the G-alone vaccine were significantly enriched in the inflammatory pathways, such as the cytokine and inflammatory response, cytokine network, and complement pathway compared to those in PBS-vaccinated mice; these effects were abolished by CD4^+^ T cell depletion (Supplementary Figs. 4, 5). Thus, we demonstrated that CD4^+^ T cells are the primary drivers of ERD induced by G protein vaccines.

### IL-13 promotes lung weight gain

**Fig. 5:**
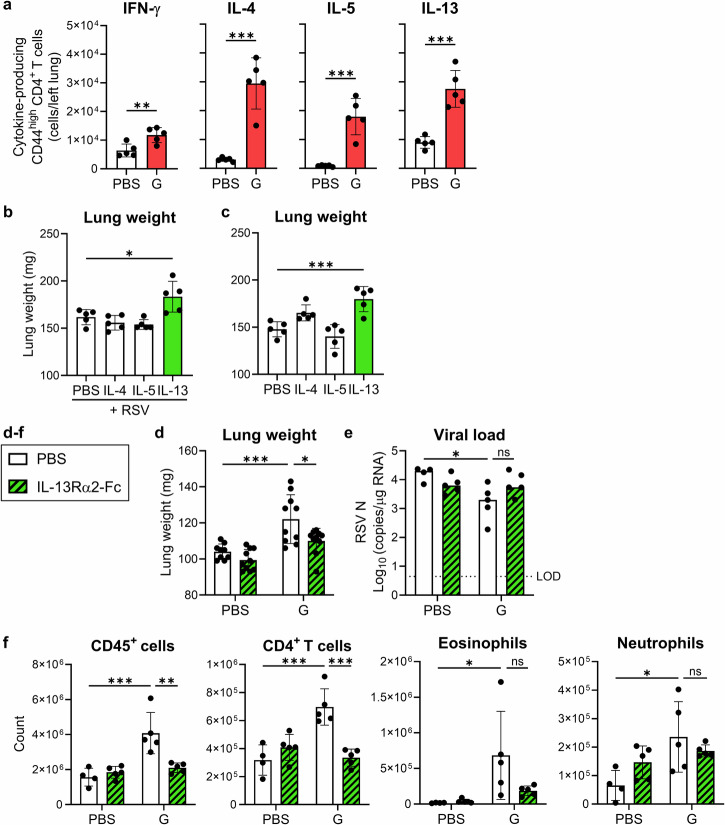
Effect of Th2 cytokines on G protein vaccine-induced lung weight gain. **a** Number of cytokine-producing CD44^high^ CD4^+^ T cells in the left lung 5 days after the RSV challenge following the G-alone vaccination. **b**, **c** Whole lung weight after daily intranasal administration of IL-4, IL-5, or IL-13 for 4 days in mice (**b**) infected or (**c**) uninfected with RSV. **d**–**f** G-alone-vaccinated mice were challenged with the RSV and analyzed on day 5 post challenge. After the RSV challenge, mice were treated with IL-13Rα2-Fc or PBS. **d** Right lung weight. **e** Viral loads in the right lungs. **f** Number of CD45^+^ cells, CD4^+^ T cells, eosinophils, and neutrophils in the left lung. **a**–**f** Each experiment was performed twice. **a**–**c**, **e**, **f** *n* = 5 per group. **d** *n* = 9–10 per group. **a**–**f** Data are presented (**a**–**d**, **f**) as the mean ± SD or (**e**) median. **a**–**f** *P < 0.05, **P < 0.01, ***P < 0.001 as indicated using (**a**) unpaired Student *t* test, (**b**, **c**) one-way ANOVA with Dunnett’s test compared with PBS, or (**d**–**f**) two-way ANOVA with Tukey’s test. ns not statistically significant. LOD limit of detection.

We examined CD4^+^ T cell phenotypes and cytokines involved in ERD using the G-alone vaccine. To investigate the phenotypes of CD4^+^ T cells infiltrating the lungs after the RSV challenge, we analyzed the CD4^+^ T cells producing Th1 cytokines (IFN-γ) or Th2 cytokines (IL-4, IL-5, and IL-13) using flow cytometry (Fig. 5a, Supplementary Fig. 6). The number of Th1 cells in the G-alone-vaccinated mice was slightly, but significantly, higher than that in the PBS-vaccinated mice (Fig. 5a). The number of Th2 cells in the former group was significantly higher than that in the latter group (Fig. 5a). The number of Th2 cells was higher than that of Th1 cells in the G-alone-vaccinated mice (Fig. 5a). In the G+Alum-vaccinated mice, the number of Th2 cells was significantly higher than that in the PBS-vaccinated mice (Supplementary Fig. 7). These findings revealed that G protein-vaccinated mice showed a remarkable increase in Th2 cells infiltrating the lungs after RSV challenge.

To identify which Th2 cytokines promote ERD, we analyzed the lungs of RSV-infected mice after intranasal administration of each Th2 cytokine. Compared to in the PBS-treated group, the IL-13-treated group showed increased lung weight while the IL-4- and IL-5-treated groups did not (Fig. 5b). In addition, intranasal administration of each Th2 cytokine to naive mice significantly increased lung weight in IL-13-treated mice compared to that in PBS-treated mice, whereas IL-4 and IL-5 did not have this effect (Fig. 5c). To verify whether lung weight gain in the G-alone-vaccinated mice is IL-13 dependent, we administered IL-13Rα2-Fc to neutralize IL-13 after the RSV challenge. In the G-alone-vaccinated mice, IL-13 neutralization significantly decreased lung weight compared to that in the PBS-treated group, although the viral load did not change (Fig. 5d, e). In addition, IL-13 neutralization significantly reduced the number of CD45^+^ and CD4^+^ T cells compared to that in the PBS-treated group (Fig. 5f). Moreover, IL-13 neutralization significantly reduced the number of Th1- and IL-4-producing Th2 cells in the lungs compared to those in the PBS-treated group (Supplementary Fig. 8). Thus, IL-13 promoted lung weight gain in mice vaccinated with G-alone and IL-13 was also involved in the migration of CD4^+^ T cells into the lungs.

### G protein vaccine exacerbates IL-13-mediated mucin hypersecretion after RSV challenge

**Fig. 6:**
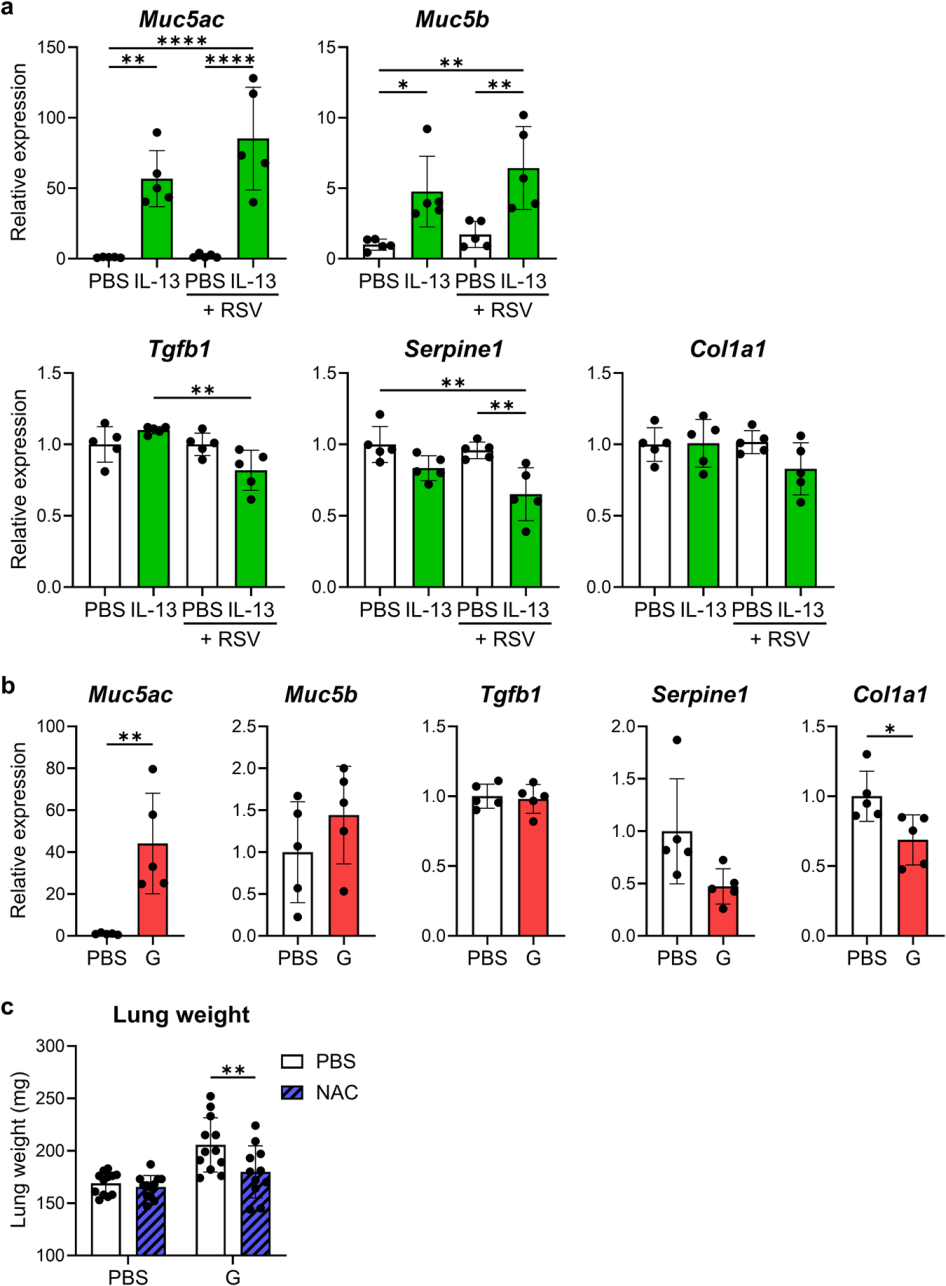
Contribution of IL-13-induced mucin production to lung weight gain. **a**, **b** Relative expression levels of *Muc5ac*, *Muc5b*, *Tgfb1*, *Serpine1*, and *Col1a1* mRNA in the right lung (**a**) after intranasal IL-13 treatment with or without RSV infection or (**b**) after RSV challenge following the G-alone vaccination. **c** Whole lung weights after N-acetyl-L-cysteine (NAC) treatment in RSV-challenged G-alone-vaccinated mice. **a**–**c** Each experiment was performed twice. **a**, **b** *n* = 5 per group. **c** n = 11–12 per group. **a**–**c** Data are presented as the mean ± SD. *P < 0.05, **P < 0.01, ****P < 0.0001 as indicated using (**a**) one-way ANOVA with Tukey’s test, (**b**) unpaired Student *t* test, or (**c**) two-way ANOVA with Tukey’s test.

IL-13 promotes mucin production and fibrosis in the lung^27–30^. To investigate how IL-13 causes lung weight gain, we analyzed the mRNA expression of mucins (*Muc5ac* and *Muc5b*) and fibrosis-related proteins (*Tgfb1*, *Serpine1*, and *Col1a1*) in the lungs of intranasally IL-13-treated mice and G-alone-vaccinated mice after the RSV challenge. Intranasal administration of IL-13 significantly increased the mRNA expression of mucin, but not that of fibrosis-related proteins, in the lungs of mice with and without the RSV infection, compared to that in the PBS-treated group (Fig. 6a). In the G-alone-vaccinated mice, the mRNA expression of *Muc5ac* was significantly increased in the lungs after the RSV challenge compared to that in PBS-vaccinated mice, whereas the mRNA expression of *Muc5b* was not (Fig. 6b). The mRNA expression of fibrosis-related proteins was similar or even decreased in the G-alone-vaccinated mice compared to that in the PBS-vaccinated mice (Fig. 6b). Therefore, the G protein vaccine may enhance IL-13-mediated mucin hypersecretion in ERD.

Finally, we examined whether the removal of mucin from the lungs reduced the lung weight. N-acetyl-l-cysteine (NAC) cleaves the disulfide bonds in mucin to remove mucin^31,32^. In mice vaccinated with the G-alone vaccine, intranasal administration of NAC after the RSV challenge significantly reduced lung weight compared to that in the PBS-treated group (Fig. 6c). Thus, we suggest that lung weight gain is associated with IL-13-mediated mucin hypersecretion following the RSV challenge in G protein-vaccinated mice.

## Discussion

We found that the G protein vaccine induced lung weight gain along with pathogenetic changes after the RSV challenge. Lung weight gain has not been reported in previous studies on G protein vaccines. Lung weight is commonly used as an indicator of tissue damage; however, the cause of lung weight gain in many pulmonary diseases is not well understood. Herein, we showed that G protein vaccine-induced lung weight gain correlated with lung pathology. In addition, we observed that the increased lung weight was reduced by intranasal administration of NAC, suggesting the possibility that the lung weight gain correlates with mucin accumulation. Because of the hydrating nature of mucin^33^, an increase in mucin may cause lung weight gain owing to an increase in the water content. However, NAC has antioxidant and anti-inflammatory effects in addition to mucin removal^34,35^. Therefore, lung weight gain may be a combined result of mucin hypersecretion and the production of other inflammatory factors induced by IL-13. Future research should elucidate the detailed causes of lung weight gain, which will further support the physiological importance of using lung weight as an indicator of ERD severity.

We elucidated the pathogenesis underlying ERD mediated by IL-13 in the recombinant G protein vaccine. Previous studies have demonstrated that in mice vaccinated with a G protein-expressing vaccinia viral vector, ERD was mediated by IFN-γ expression rather than by IL-13, and this was characterized by symptoms such as weight loss and increased airway resistance^36,37^. Our findings differ from these results in the following ways. First, we found that ERD was mediated by IL-13 and mucin hypersecretion; however, we agree with their conclusion that eosinophils are not involved in ERD. Second, the recombinant G protein vaccine and G protein-expressing vaccinia viral vector-based vaccines have different immunological properties. In general, vaccinia viral vector-based vaccines are potent inducers of Th1 cells. Other studies have shown that G protein-expressing vaccinia viral vector-based vaccines increase the expression levels of *Ifng* mRNA following RSV challenge to a degree comparable to *Il13* mRNA expression in the lungs^38^. Meanwhile, our results showed that the recombinant G protein vaccine induced fewer Th1 cells and more Th2 cells. In our previous report, the recombinant G protein vaccine minimally increased Th1 cytokine mRNA expression levels but significantly increased Th2 cytokine mRNA expression levels in the lungs after RSV challenge^12^. As the recombinant G protein vaccine and vaccines based on G protein-expressing vaccinia viral vectors induce different immune responses, the mechanism of ERD induced by these vaccines is likely to be different and needs to be distinguished.

We demonstrated that lung weight gain and lung pathogenesis were triggered by IL-13 via Th2 cell infiltration into the lung after the RSV challenge. RNA-seq showed that the expression of Th2 cytokine genes, particularly IL-13 and IL-13-related genes, was high in the lungs of G protein-vaccinated mice, suggesting an inflammatory response primarily comprising IL-13. In addition, IL-13 acts on goblet cells and increases the expression of the secretory mucin MUC5AC^21,39^. Indeed, pathological staining showed goblet cell hyperplasia and mucin hypersecretion in the lungs of G protein-vaccinated mice, whereas RNA-seq and real-time RT-PCR showed a significant increase in *Muc5ac* mRNA expression. These results suggest that the levels of MUC5AC in the lungs may correlate with lung weight gain. In addition, IL-13 neutralization after the RSV challenge inhibited lung weight gain and the infiltration of CD4^+^ T cells into the lungs. IL-13 promotes the expression of CCL17 and CCL22^40,41^, which are involved in the migration of Th2 cells. The migration of Th2 cells into the lungs causes IL-13 production in the lungs^42^. Therefore, Th2 cells may create a positive feedback loop to further recruit Th2 cells via IL-13, and accelerate mucin production and lung weight gain^43^. Currently approved recombinant F protein vaccines, consisting of the F protein alone or with a Th1-type adjuvant, have not been reported to induce ERD in clinical trials^6–8,44^. In contrast, in animal models, the recombinant F protein vaccine combined with Th2-type adjuvants, such as Alum or AddaVax, induces Th2 cell production and exacerbates lung pathology^45–47^. Therefore, the induction of Th2 cells should be carefully validated in F protein vaccines to prevent ERD induction.

We found that the vaccine comprising G protein alone did not induce detectable G-specific IgG; however, it did induce the production of Th2 cells, resulting in mucin hypersecretion and lung weight gain. Previous reports have suggested that the G-specific monoclonal IgG 131-2 G, which exhibits neutralizing activity^48^, is more effective at reducing the RSV infection-induced excessive mucin production in naive mice (compared with an F-specific monoclonal IgG)^49,50^. Therefore, G-specific neutralizing antibodies may contribute to the prevention of ERD. Meanwhile, immune complex formation and complement activation following the binding of non-neutralizing antibodies to RSV have been suggested as causes of ERD after FI-RSV vaccination^51,52^. Although it is possible that Th2 cells and non-neutralizing antibodies cooperate to exacerbate ERD, our results indicate that Th2 cells are the primary cause of ERD induction.

We demonstrated that eosinophils did not affect lung pathogenesis and mucin hypersecretion in G protein-vaccinated mice. Eosinophils are known to cause airway hyperresponsiveness during RSV infection in naive mice^53,54^. In addition, the severity of ERD after FI-RSV vaccination has been reported to correlate with the number of eosinophils in the lungs^55^. Therefore, the number of eosinophils has been used as an indicator of ERD severity in G protein vaccines in many studies^10,15,36,56,57^. Furthermore, Th2 cells and IL-13 are responsible for the induction of eosinophil infiltration into the lung after RSV challenge in G protein-vaccinated mice^36,56,57^. However, some reports have suggested that eosinophils are not involved in ERD induction^37,58^. This is because, even in eosinophil-deficient mice, the G protein vaccine or FI-RSV vaccine mediates increased airway hyperresponsiveness and decreased respiratory function as observed in wild-type mice^37,58^. In addition, in eosinophil-deficient mice, the FI-RSV vaccine induces the same level of mucin secretion as in the wild type^58^, consistent with our results that the eosinophil depletion did not affect lung pathogenesis and mucin hypersecretion induced by the G protein vaccine. Taken together, the present and previous studies have suggested that eosinophils are involved in exacerbating airway hyperresponsiveness during RSV infection in naive mice, whereas they are not involved in lung pathogenesis and mucin hypersecretion in ERD. Therefore, our results suggest that eosinophils are not appropriate indicators of ERD severity in G protein vaccines.

Although eosinophils did not affect the ERD, they did contribute to protection against RSV. Previous reports have suggested that eosinophils are involved in RSV elimination via the production of type I IFN and nitric oxide^59^, and our findings are consistent with this. In the present study, neutrophil depletion with anti-Ly6G antibody significantly increased viral load and significantly decreased the number of eosinophils following RSV challenge in G+Alum-vaccinated mice. A subset of eosinophils expresses Ly6G^60,61^. In this study, some eosinophils infiltrating the lungs after the RSV challenge of G protein-vaccinated mice expressed low to moderate levels of Ly6G, and these eosinophils were also depleted with anti-Ly6G antibodies. Therefore, the decrease in Ly6G-expressing eosinophils may be responsible for the increase in viral load, owing to neutrophil depletion. In addition, it is possible that neutrophils eliminate RSV. Although unknown in RSV infection, neutrophils may directly protect against influenza virus infection^62,63^. In addition, eosinophils generally migrate into tissues via IL-5 and IL-13 produced by Th2 cells and type 2 innate lymphoid cells^64^, and neutrophils are involved in eosinophil migration in vitro^64,65^. Therefore, neutrophils may contribute to RSV clearance either directly or indirectly by promoting eosinophil migration into the lung.

Because Th2 cell-mediated IL-13 induces ERD, we propose that for the development of safe G protein vaccines, it is important to design vaccines that do not induce Th2 cells by optimizing the adjuvant or vaccine antigen. We previously reported that the addition of CpG oligodeoxynucleotides, a Th1-type adjuvant, to recombinant G protein vaccines did not induce lung weight gain^12^. Regarding the optimization of vaccine antigens, the G protein has the potential to induce Th2 cell production^10,12,52^; therefore, it is necessary to modify the regions of the G protein that are involved in the induction of Th2 cells. A large number of O-linked glycans are added to G proteins^1^. We previously showed that a vaccine containing a recombinant G protein produced in *Escherichia coli*, which does not have a glycosylation system, is less likely to induce G-specific Th2 cells and lung weight gain than vaccines containing recombinant G proteins produced in mammalian cells^12^. However, it is not clear whether the difference in the immune response between the two G proteins can be attributed to the glycans or their conformation. In addition, we have demonstrated that the combination of CpG oligodeoxynucleotides and recombinant G protein produced using *E. coli* can further improve safety and optimize both the adjuvant and vaccine antigen^12^. This evidence may help elucidate the reason for the previously reported improved safety of G protein vaccines.

ERD has been reported in animal models not only with RSV vaccines, including the FI-RSV vaccine, but also with the subunit vaccine against severe acute respiratory syndrome coronavirus 2 (SARS-CoV-2) and the inactivated virus vaccine against the Middle East respiratory syndrome coronavirus (MERS-CoV)^66–68^. Although these vaccines are known to induce Th2-type immune responses, the association of IL-13 and mucin with pulmonary immunopathology has not been studied previously, and the mechanism of ERD remains unknown. Therefore, our results are expected to provide useful insights into the causes of ERD and clinical applications of these vaccines.

## Methods

### Mice

Six-week-old female BALB/c mice were purchased from SLC (Hamamatsu, Shizuoka, Japan). They were housed under a 12-hour light/dark cycle (lights on at 8:00 AM, lights off at 8:00 PM) with *ad libitum* access to food and water. All animal procedures were approved by the Animal Care and Use Committee of the Research Institute for Microbial Diseases, Osaka University, Japan (protocol numbers: BIKEN-AP-R01-15-2 and BIKEN-AP-R02-14-5) and were performed according to Osaka University’s Institutional Guidelines for the Ethical Treatment of Animals.

### Production and purification of recombinant G protein and IL-13Rα2-Fc protein

For G protein expression, the G protein sequence was derived from RSV (strain A2) (GenBank accession number: AAB59857.1). The plasmid vector was encoded using the human codon-optimized sequence of the human Igκ signal peptide (METDTLLLWVLLLWVPGSTGD), hexahistidine tag (His-tag), linker (GAGGG), and G protein ectodomain (amino acids 67–298). For expression of IL-13Rα2-Fc protein, the plasmid vector was encoded with the human codon-optimized sequence of human Igκ signal peptide, the extracellular domain of mouse IL-13Rα2 (GenBank accession number: AAC33240.1) (amino acids 22–334), G4S linker (GGGGS), His-tag, G4S linker, and the hinge-CH2-CH3 regions of human IgG1 (GenBank accession number: AAC82527.1) (amino acids 100–330). Recombinant G and IL-13Rα2-Fc proteins were expressed in Expi293F cells (Thermo Fisher Scientific, Hampton, NH, USA) and purified by immobilized metal affinity chromatography and size exclusion chromatography, as previously reported^12^.

### RSV progression and enrichment

RSV (strain A2) was produced using HEp-2 cells as previously reported^12^. Briefly, for RSV progression, one plaque forming unit (PFU) of RSV per 10^2^ HEp-2 cells was added to 70–80% confluent HEp-2 monolayers and incubated at 37 °C with 5% CO_2_ for 7 h with shaking every hour. The culture medium was then replaced with fresh medium. After incubation at 37 °C with 5% CO_2_ for 5 days, the cells were freeze-thawed to collect the virus, and the supernatant was collected by centrifugation twice at 700 × *g* for 5 min at 4 °C. For RSV enrichment, the supernatant was transferred to an ultracentrifuge tube, to which 1 mL of PBS containing 20% sucrose was added, and centrifuged at 71,000 × *g* for 3.5 h at 4 °C. The precipitated virus was resuspended in PBS, aliquoted, and stored at –80 °C until use. Virus titers were measured using a plaque assay as previously reported^12^. Briefly, the virus diluent was added to 90% confluent HEp-2 monolayers and incubated at 37 °C with 5% CO_2_ for 2 h. Following the incubation, the culture medium was replaced with fresh medium containing 0.6% carboxymethylcellulose. Subsequently, the cells and viruses were fixed with methanol after incubation at 37 °C with 5% CO_2_ for 3 days. The fixed viruses were then treated with goat anti-RSV polyclonal antibody (catalog number: AB1128, dilution 1/500; Merck Millipore, Darmstadt, Germany) and horseradish peroxidase-conjugated donkey anti-goat IgG polyclonal antibody (catalog number: A15999, dilution 1/150; Thermo Fisher Scientific). The plaques were visualized using 4-chloro-1-napthol (Tokyo Chemical Industry Co., Ltd., Tokyo, Japan) and subsequently counted. All animal experiments were reviewed and approved by the Institutional Review Board of the Research Institute for Microbial Diseases, Osaka University (protocol number: BIKEN-00224-002).

### Vaccination and RSV challenge

Mice were vaccinated subcutaneously with 1 μg of G protein, either alone or with 50 μg of Alhydrogel adjuvant 2% (Alum; InvivoGen, San Diego, CA, USA), in 50 μL of PBS at the base of the tail on days 0 and 21. On day 28, plasma samples were collected from the cheek vein to assess G-specific IgG levels. Following euthanasia via cervical dislocation, spleens were harvested for the T-cell restimulation assay, as previously described^12^. On day 31, mice were intranasally challenged with 1.0 × 10^5^ PFU of RSV in 30 μL of PBS (15 μL to each nostril) under anesthesia. After RSV challenge, the mice were anesthetized and euthanized. Lungs and BALF samples were collected for use in subsequent assays. In the experiments shown in Figs. 1d, 2a, 2d, 3a, 3d, and 5d, the weight of the right lung was measured. In the experiments shown in Figs. 5b, 5c, and 6c, the weight of both lungs was measured.

### Detection of antibodies and cytokines

Plasma levels of G-specific IgG were detected using an indirect enzyme-linked immunosorbent assay (ELISA), as previously reported^12^. Briefly, 96-well half-area flat-bottom plates (Corning, NY, USA) were coated with 1 μg/mL G protein in carbonate buffer. Diluted plasma was added to G protein-coated wells. Antibodies bound to G protein were incubated with the antibodies listed in Supplementary Table 1. The coloration reaction was performed using tetramethylbenzidine, followed by stopping with 2 N H_2_SO_4_. The difference in optical density (OD) between 450 and 570 nm (OD_450-570_) was detected using a microplate reader (Power Wave HT; BioTek, Winooski, VT, USA). Cytokine levels were detected using a sandwich ELISA with the reagents listed in Supplementary Table 1. IL-13 levels were determined according to the manufacturer’s instructions using an IL-13 Mouse Uncoated ELISA Kit (Thermo Fisher Scientific). OD_450-570_ was measured using a microplate reader.

### Detection of mRNA expressions

The right lungs were collected in 1 mL TRIzol Reagent (Thermo Fisher Scientific) and homogenized with three 4 mm stainless steel beads (TAITEC, Saitama, Japan) using a bead beater-type homogenizer (Beads Crusher μT-12; TAITEC) for 60 s at 3200 rpm. RNA was purified using TRIzol Reagent according to the manufacturer’s instructions. Reverse transcription was performed using ReverTra Ace qPCR RT Master Mix (Toyobo, Osaka, Japan) to synthesize cDNA. Real-time RT-PCR was performed by amplifying the target mRNA and *Gapdh* mRNA as a reference gene using a Light Cycler 480-II (Roche Diagnostics, Tokyo, Japan) and LightCycler 480 SYBR Green I Master (Roche Diagnostics), with the primers listed in Supplementary Table 2. The absolute levels of RSV were calculated by amplifying the plasmid encoding the RSV nucleoprotein (RSV N) gene and generating a standard curve. Relative mRNA expression levels were calculated by dividing the target mRNA expression levels by *Gapdh* mRNA expression levels, with the mean value of the control group expressed as 1.

### LDH assay

BALF (1 mL) was centrifuged at 600 × *g* for 5 min, and LDH levels in the supernatant were measured using the Cytotoxicity LDH Assay Kit-WST (Dojindo, Kumamoto, Japan) according to the manufacturer’s instructions. OD at 490 nm (OD_490_) using a microplate reader.

### Analysis of infiltrating cells into the lungs

Cells infiltrating the lungs were analyzed as previously reported^12^. Briefly, the left lungs were sheared and digested using collagenase IV and DNase I. Then, the lungs were dissociated and hemolyzed. For surface antigen staining, lung cells were labeled with the Fixable Viability Dye eFluor 780 (catalog number: 65-0865-18, dilution 1/1000; Thermo Fisher Scientific), and the antibodies are listed in Supplementary Table 3. Flow cytometric analysis was performed using an Attune NxT Flow Cytometer (Thermo Fisher Scientific), and data were analyzed using FlowJo software version 10.10 (FlowJo LLC, Ashland, Oregon, USA).

### Depletion of eosinophils, neutrophils, and CD4^+^ T cells

Mice were vaccinated with the G protein, with or without alum, on days 0 and 21. On day 31, mice were challenged with RSV. For the depletion of CD4^+^ T cells, mice were injected intraperitoneally on day 30 with 100 μg of rat anti-mouse CD4 antibody (clone: GK1.5, catalog number: BE0003-1; Bio X Cell, West Lebanon, NH, USA) or rat IgG2b isotype control (clone: LTF-2, catalog number: BE0090; Bio X Cell) in 200 μL PBS. For the depletion of eosinophils, mice were injected intraperitoneally on day 30 and 33 with 100 μg of rat anti-mouse CCR3 antibody (clone: 6S2-19-4, catalog number: BE0316; Bio X Cell) or rat IgG2b isotype control in 200 μL PBS. For the depletion of neutrophils, mice were injected intraperitoneally on day 30, 32, and 34 with 50 μg of rat anti-mouse Ly6G antibody (clone: 1A8, catalog number: 127649; BioLegend) or rat IgG2a isotype control (clone: RTK2758, catalog number: 400565; BioLegend) in 200 μL PBS. The right lungs were weighed and collected to measure the viral load. The left lung was collected for analysis of infiltrating cells.

### Histopathology analysis

The excised lungs were fixed by injecting 10% phosphate-buffered formalin into the trachea until the lungs were inflated. The left lung of the fixed lung was then embedded in paraffin, sectioned, and stained with H&E or PAS. The histopathological scoring method utilized an ordinal scale with “1” indicating normal or naive status and “4” indicating extensive or severe status, as previously reported^58^. Specific scoring criteria were as follows: PVA: 1, normal, within naive parameters; 2, focal to uncommon numbers of solitary cells with uncommon aggregates; 3, multifocal moderate aggregates; 4, moderate to high cellularity and multifocal, large cellular aggregates that may be expansive into adjacent tissues. Specific scoring criteria were as follows: mucin: 1, no mucin; 2, goblet cell hyperplasia with none to rare luminal mucin; 3, goblet cell hyperplasia with luminal mucin accumulation in airways; 4, severe mucin alterations, with some airways completely obstructed by mucin.

### RNA-seq

Total RNA was extracted from the right lung using a QuickGene-AutoS RNA cultured cell kit (Kurabo, Osaka, Japan) according to the manufacturer’s instructions. RNA libraries were prepared using a TruSeq Stranded mRNA Library Prep Kit (Illumina, San Diego, CA, USA) according to the manufacturer’s instructions. Sequencing was performed on NovaSeq 6000 platform in a 101-base single-end mode. RTA v3.4.4 software (Illumina) was used for base calling. Generated reads were mapped to the mouse (mm10) reference genome using TopHat v2.1.1 in combination with Bowtie2 ver. 2.2.8 and SAMtools ver. 0.1.18. Fragments per kilobase of exon per million mapped fragments (FPKMs) were calculated using Cuffdiff 2.2.1 with parameter-max-bundle-frags 50,000,000. Principal component analysis, heatmap clustering, and volcano plot analysis were performed using iDEP 2.01 (http://bioinformatics.sdstate.edu/idep/). Gene set enrichment analysis was performed using GSEA v4.3.3. Dot plots of gene set enrichment analysis were generated using the R function ggplots2. The RNA-seq data concerning this study have been deposited in the Gene Expression Omnibus (GEO) under the accession number GSE272499.

### Analysis of T cells infiltrating the lung

The cells isolated from the left lungs (1–3 × 10^6^ cells/well) were cultured in Roswell Park Memorial Institute 1640 medium with 10% fatal bovine serum (FBS), 1% penicillin, 1% streptomycin, and 50 µM 2-mercaptoethanol. The cells were stimulated with 100 ng/mL phorbol 12-myristate 13-acetate and 2 μg/mL ionomycin in presence of protein transport inhibitor cocktail (Thermo Fisher Scientific) for 4 h at 37 °C with 5% CO_2_ in 96-well U-bottom plates. For surface antigen staining, cells were incubated with Fixable Viability Dye eFluor 780, and the antibodies are listed in Supplementary Table 3. Subsequently, for intracellular cytokine staining, the cells were permeabilized and incubated with the antibodies (Supplementary Table 3) using the BD Cytofix/Cytoperm Fixation/Permeabilization Solution Kit (BD Biosciences, Sparks, MO, USA) according to the manufacturer’s instructions. Flow cytometric analysis was performed using an Attune NxT Flow Cytometer, and data were analyzed using FlowJo software version 10.10.

### Intranasal administration of Th2 cytokines

Mice infected or uninfected intranasally with 1.0 × 10^5^ PFU of RSV on the previous day were intranasally administered 1 μg of mouse IL-4 (BioLegend), mouse IL-5 (BioLegend), or mouse IL-13 (BioLegend) in 30 μL PBS (15 μL for each nostril) under anesthesia daily for 4 days. The mice were anesthetized and euthanized on the day after the last dose. The whole lungs were weighed, and the right lungs were collected to measure the mRNA expression levels.

### IL-13 neutralization

Mice were vaccinated with G protein on days 0 and 21. On day 31, the mice were challenged intranasally with 1.0 × 10^5^ PFU of RSV in 30 μL of PBS (15 μL to each nostril) under anesthesia. On days 32, 34, and 35, the mice were injected intraperitoneally with 100 μg of IL-13Rα2-Fc in 200 μL PBS. On day 36, mice were anesthetized and euthanized. The right lungs were weighed and collected to measure the viral load. The left lung was collected for analysis of infiltrating cells.

### Intranasal administration of NAC

Mice vaccinated with the G protein alone were intranasally administered 1 mg of NAC dissolved in 30 μL PBS (15 μL in each nostril) under anesthesia on day 5 after the RSV challenge. On the day after NAC administration, the mice were anesthetized and euthanized, and their whole lungs were weighed.

### Statistical analysis

Statistical analyses were performed using the GraphPad Prism 10 software (GraphPad Software, San Diego, CA, USA). Data are expressed as the means ± standard deviation (SD) or as medians. Significant differences were determined using unpaired Student’s *t* test, one-way ANOVA with Dunnett’s test or Tukey’s test, or two-way ANOVA with Sidak’s test or Tukey’s test. Statistical significance was defined as *P* < 0.05.

## Supplementary information

Supplemental Information

## Data Availability

The findings of this study are supported by the data available in both the article and the supplementary materials. Please contact lead researcher Yasuo Yoshioka (y-yoshioka@biken.osaka-u.ac.jp) for additional information or requests concerning resources and reagents.
