# Supplementary material for: Recombinant RSV G protein vaccine induces enhanced respiratory disease via IL-13 and mucin overproduction
Source: NPJ Vaccines. 2024 Oct 12;9:187. doi: 10.1038/s41541-024-00987-w (PMC11470036; doi:10.1038/s41541-024-00987-w)
Supplement: Supplementary file 1 — Supplemental Information [file 41541_2024_987_MOESM1_ESM.pdf]

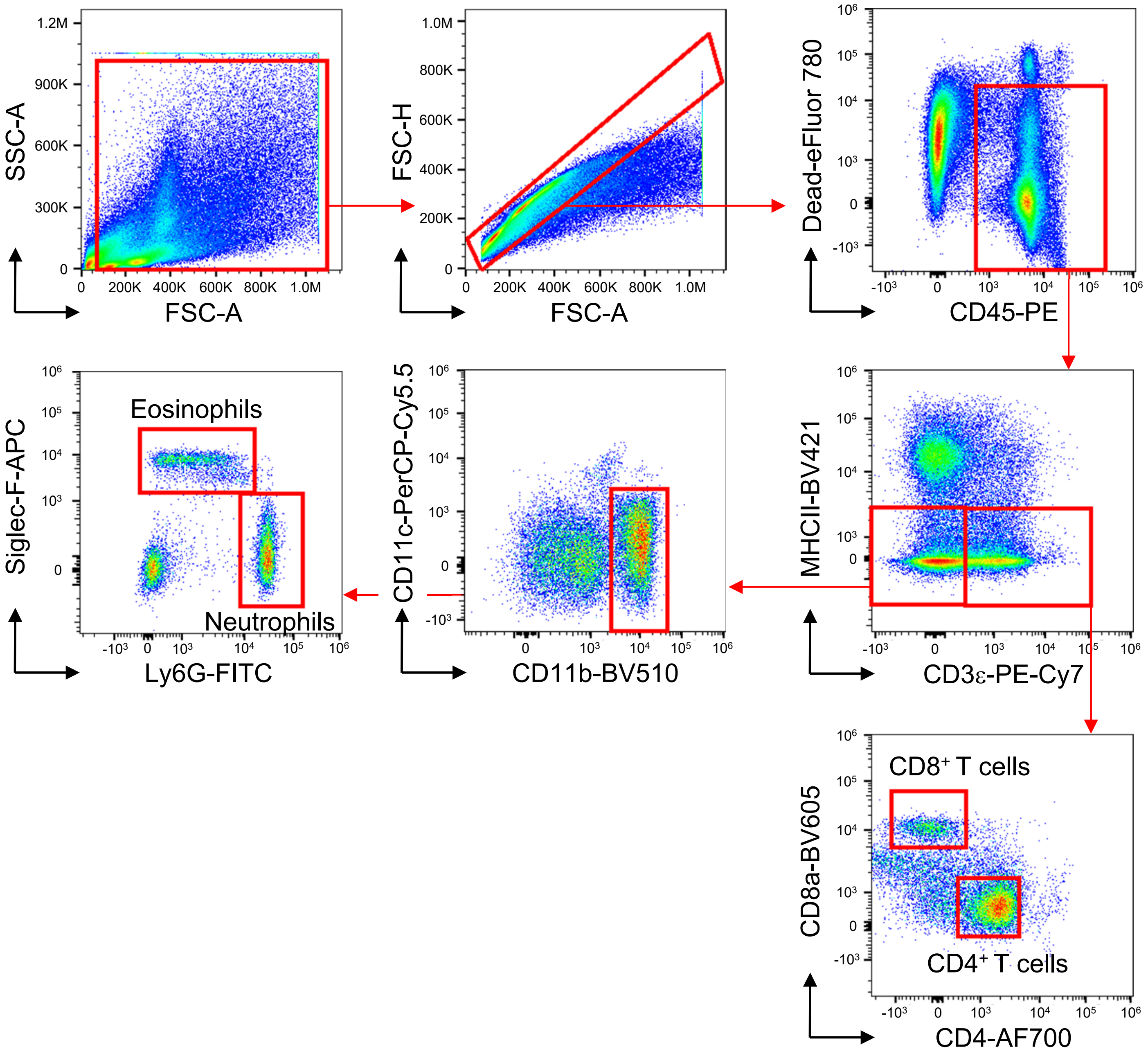

Supplementary Fig. 1. Gating strategy for infiltrating cells into the lungs after the RSV challenge following G protein vaccination.

Supplementary Figure 1. Kawahara et al

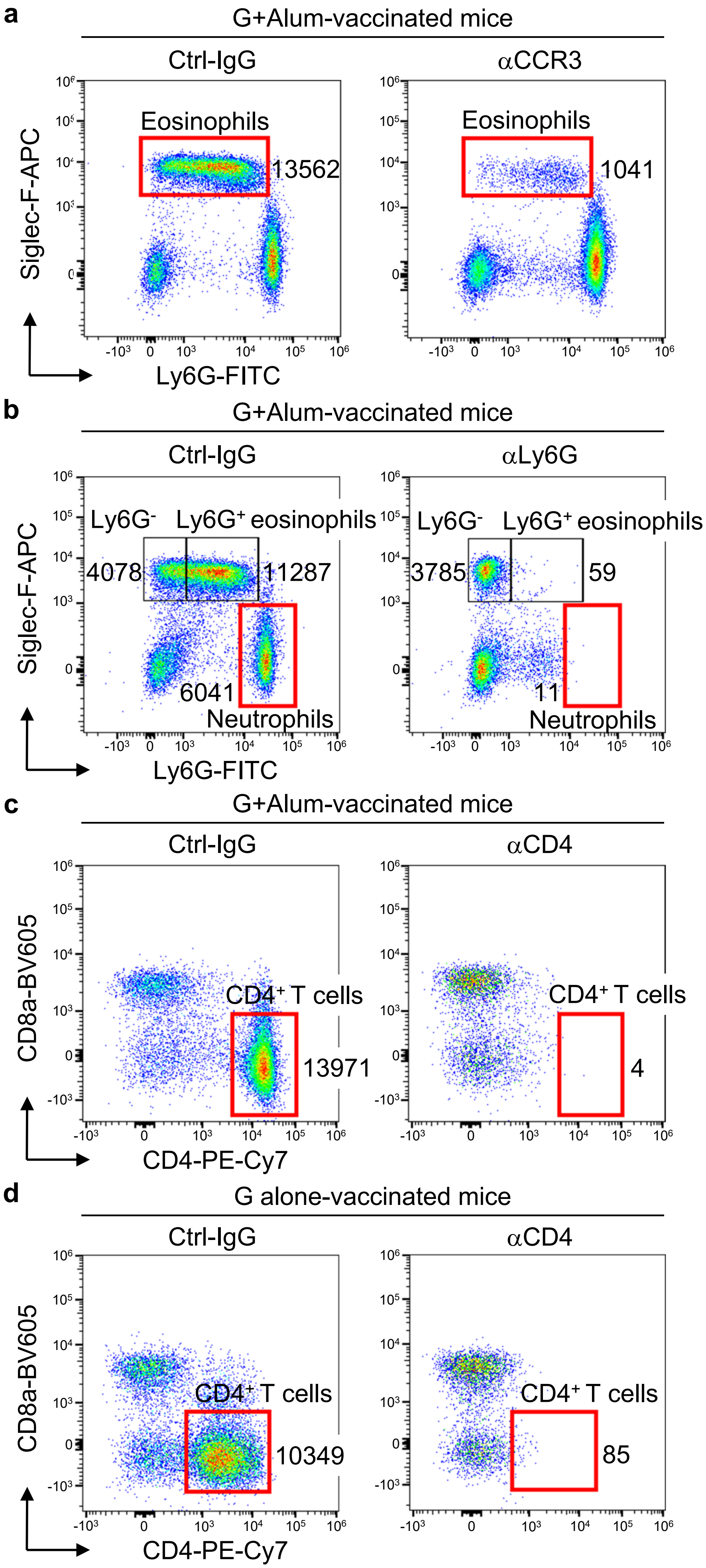

Supplementary Fig. 2. Efficiency of cell depletion after antibody administration. (a–d) Representative dot plots in the left lung after RSV challenge in mice vaccinated with (a–c) G+Alum or (d) G-alone treated with IgG isotype control (Ctrl-IgG) or (a) anti-CCR3 antibody (αCCR3), (b) anti-Ly6G antibody (αLy6G), (c, d) anti-CD4 antibody (αCD4). (a–d) The numbers shown in the plots reflect the number of cells in the gate. Each experiment was performed twice.

Supplementary Figure 2. Kawahara et al

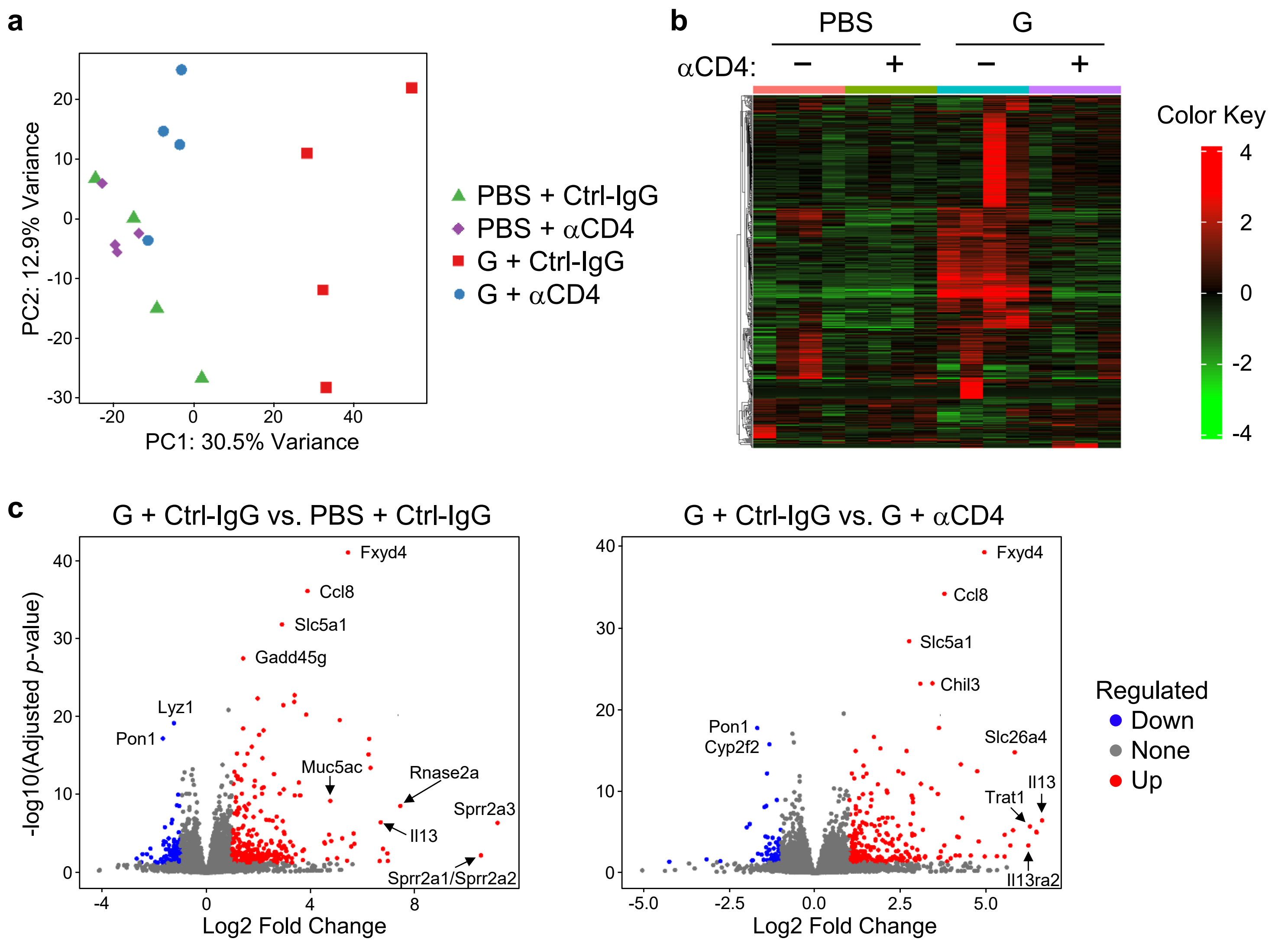

Supplementary Fig. 3. Transcriptional signatures in the lungs following the RSV challenge in G protein-vaccinated mice. (a–c) Mice vaccinated with the G-alone vaccine were treated with anti-CD4 antibody (αCD4) or IgG2b isotype control (Ctrl-IgG) prior to RSV challenge. The lungs were analyzed using RNA sequencing (RNA-seq) on day 5 post-challenge. (a) Principal component analysis of differentially expressed genes (DEGs). (b) Heatmap showing levels of expression of the top 500 DEGs. Color coding shows the z-score values of each sample as indicated in the scale, with red above the mean and green below the mean. (c) Volcano plot comparing DEGs from the lungs of PBS-vaccinated mice with Ctrl-IgG treatment and G-alone-vaccinated mice with αCD4 treatment (left) or DEGs from the lungs of G-alone-vaccinated mice with Ctrl-IgG and αCD4 treatment (right). Red and blue indicate upregulated and downregulated genes, respectively, with a fold change > 2 and a false discovery rate (FDR) < 0.05. (a–c) n = 4 per group.

Supplementary Figure 3. Kawahara et al

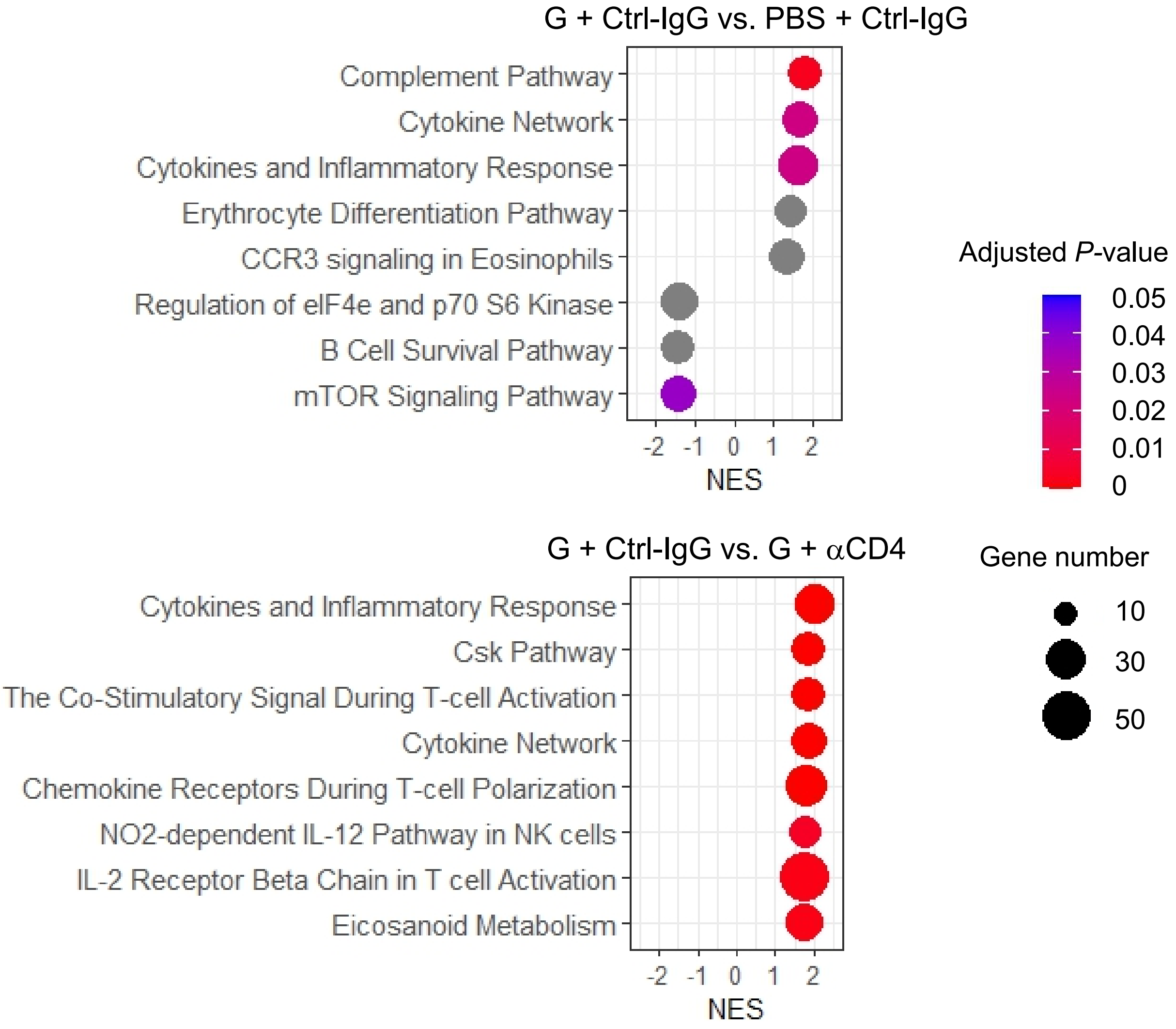

Supplementary Fig. 4. Gene set enrichment analysis of the lungs following the RSV challenge in G protein-vaccinated mice. Mice vaccinated with the G-alone were treated with anti-CD4 antibody (αCD4) or IgG2b isotype control (Ctrl-IgG) prior to RSV challenge. The lungs were analyzed using RNA-seq on day 5 after the challenge. Gene set enrichment analysis comparing DEGs from the lungs of PBS- and G-alone-vaccinated mice with Ctrl-IgG treatment (upper) or DEGs from the lungs of G-alone-vaccinated mice with Ctrl-IgG and αCD4 treatment (lower). Top eight gene sets for absolute normalized enrichment score (NES). The color of the dots indicates the adjusted *P*-value for each enriched pathway, with gray dots indicating the adjusted *P*-value > 0.05. The size of the dots reflects the number of genes enriched for each pathway. n = 4 per group.

Supplementary Figure 4. Kawahara et al

G + Ctrl-IgG vs. PBS + Ctrl-IgG

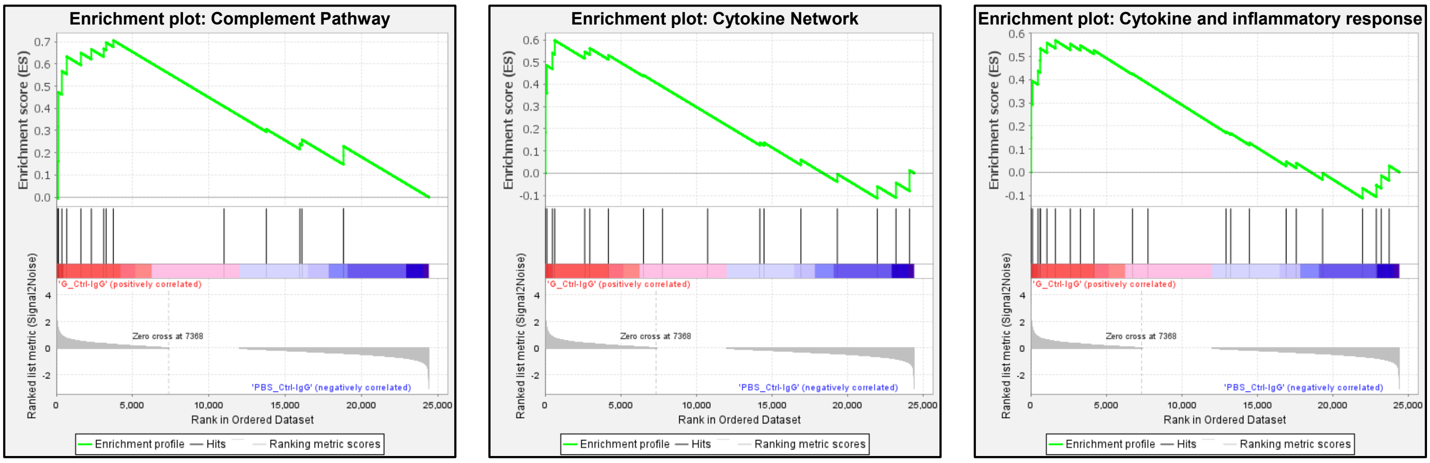

G + Ctrl-IgG vs. G + αCD4

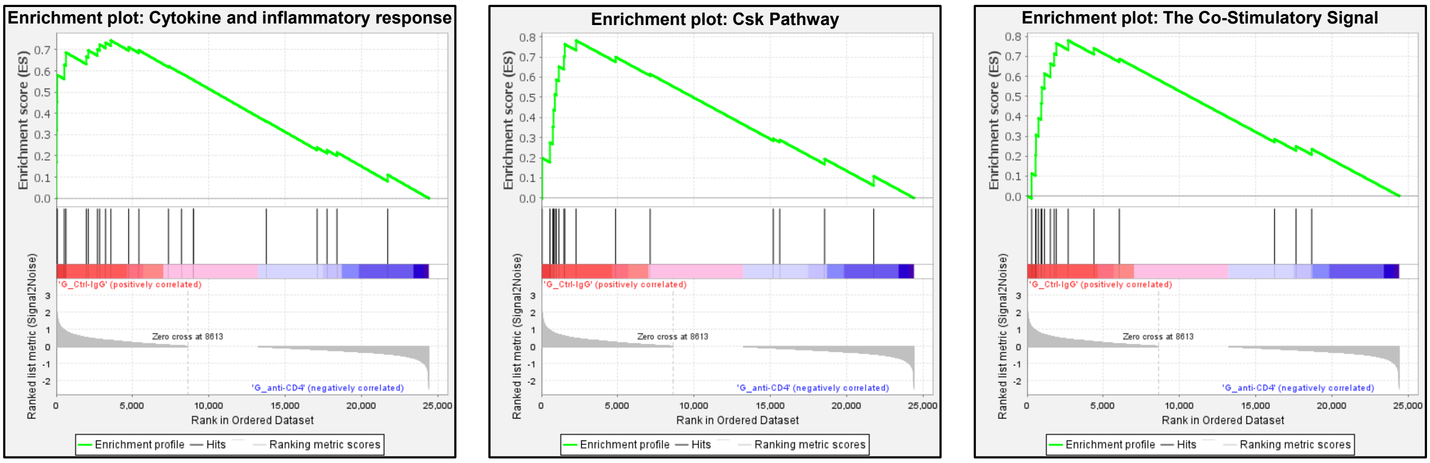

Supplementary Fig. 5. Enrichment plots of the top three sets of genes with an absolute NES in Supplementary Fig. 4.

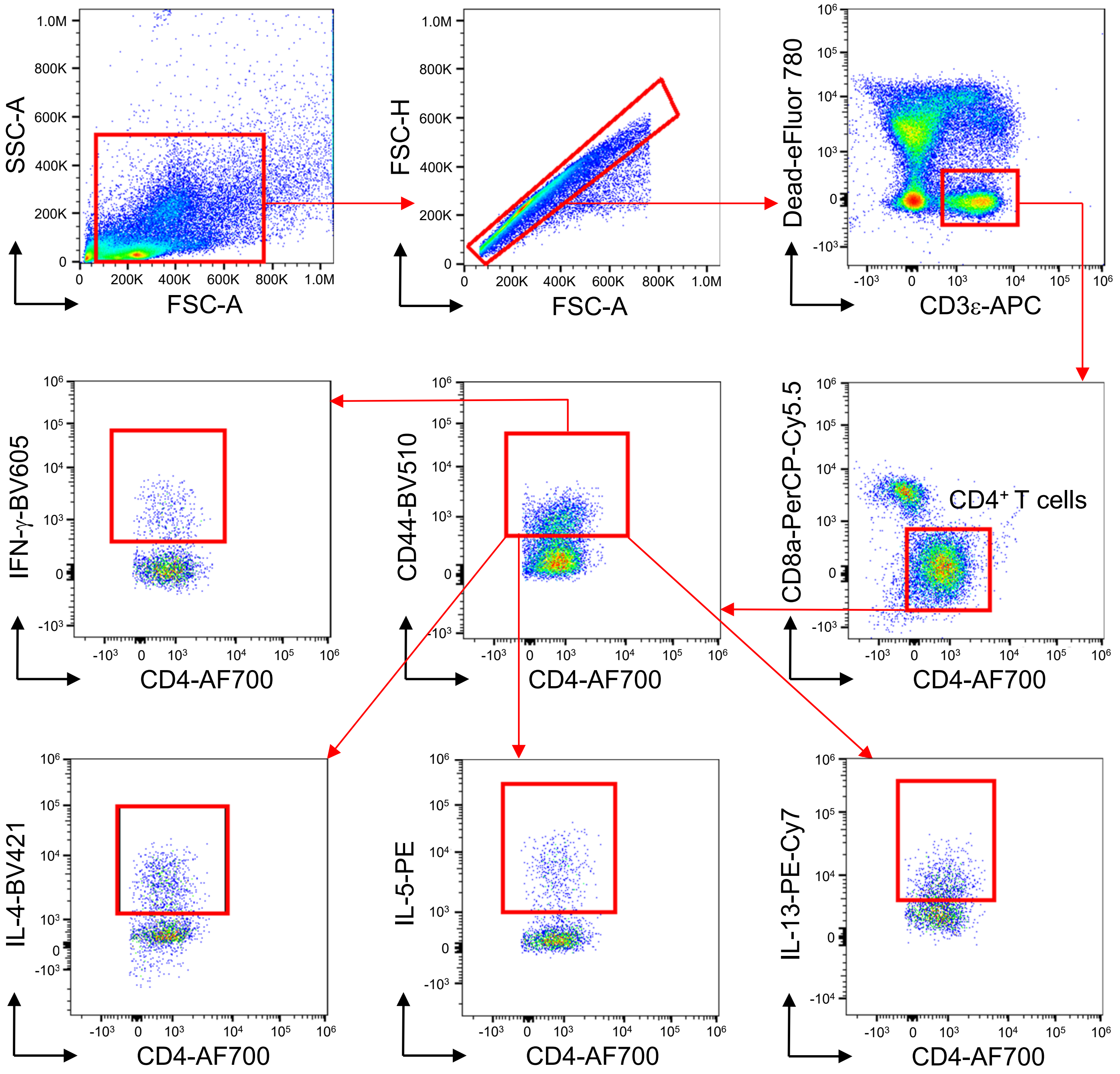

Supplementary Fig. 6. Gating strategy for cytokines producing CD44^{high} CD4^{+} T cells in the lungs after the RSV challenge following G protein vaccination.

Supplementary Figure 6. Kawahara et al

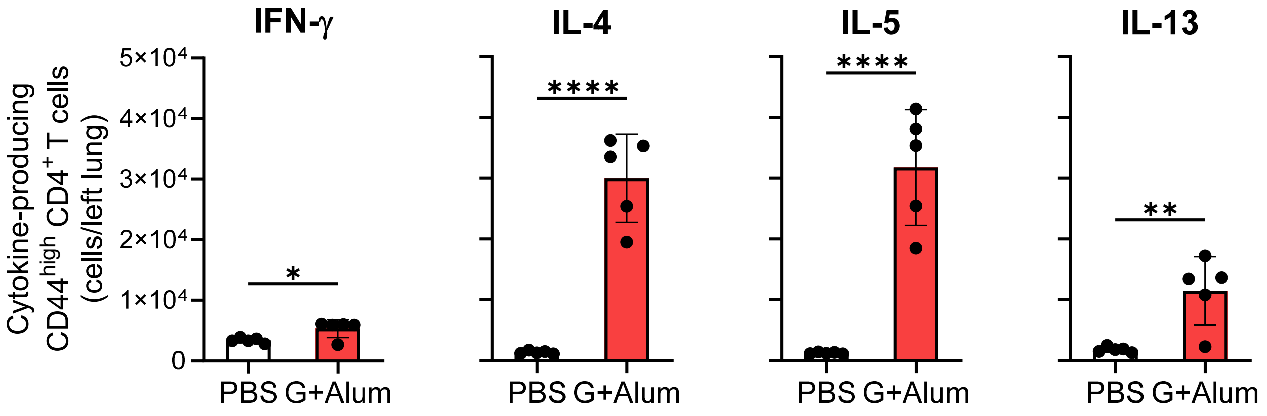

Supplementary Fig. 7. Phenotypes of $CD4^+$ T cells infiltrating into the lung after RSV challenge following G+Alum vaccination. Number of cytokine-producing $CD44^{high}$ $CD4^+$ T cells in left lung after the RSV challenge following G+Alum vaccination. The experiment was performed twice. n = 5 per group. Data are presented as the mean ± SD. $^{*}P < 0.05$, $^{**}P < 0.01$, $^{****}P < 0.0001$ as indicated using unpaired Student *t* test.

Supplementary Figure 7. Kawahara et al

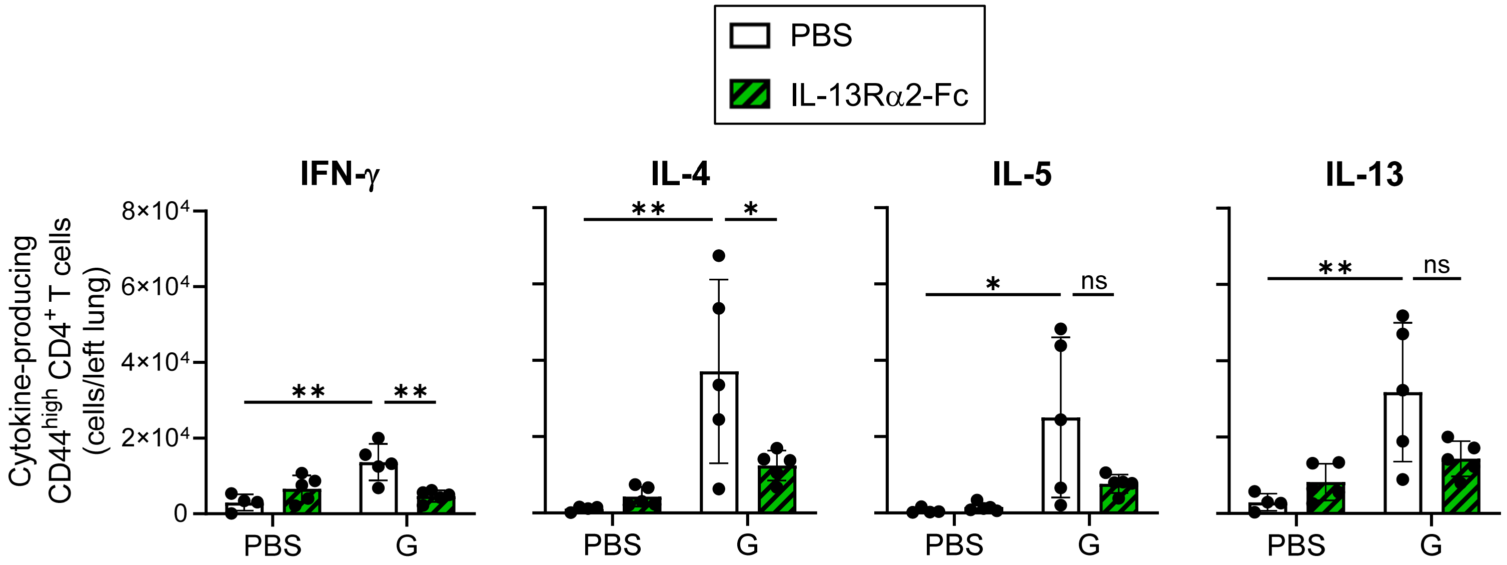

Supplementary Fig. 8. Phenotypes of $CD4^+$ T cells infiltrating into the lung with or without IL-13Rα2-Fc treatment after the RSV challenge following G-alone vaccination. Number of cytokine-producing $CD44^{high}$ $CD4^+$ T cells in left lung of mice with or without IL-13Rα2-Fc treatment after RSV challenge following G-alone vaccination. The experiment was performed twice. n = 5 per group. Data are presented as the mean ± SD. $^*P < 0.05$ and $^{**}P < 0.01$ as indicated two-way ANOVA with Tukey's test. ns, not statistically significant.

Supplementary Figure 8. Kawahara et al

Antibody	Clone	Catalog number	Dilution	Source
**Antibody measurement**				
Goat anti-mouse IgG γ chain antibody, HRP conjugate		AP503P	1:5,000	Merck Millipore
**Cytokine measurement**				
Purified anti-mouse IFN-γ antibody	R4-6A2	505701	1:250	BioLegend
Biotin anti-mouse IFN-γ antibody	XMG1.2	505803	1:250	BioLegend

Other reagent	Catalog number	Dilution	Source
HRP Streptavidin	405210	1:3,000	BioLegend

Supplementary Table 1. Antibodies and reagents used for ELISA.

Gene	5′ primer	3′ primer
RSV-N	5′-CATCCAGCAAATACACCATCCA-3′	5′-TTCTGCACATCATAATTAGGAGTATCAA-3′
Gapdh	5′-CAGGTTGTCTCCTGCGACTT-3′	5′-AGCCGTATTCATTGTCATACCAGG-3′
Muc5ac	5′-CCATGCAGAGTCCTCAGAACAA-3′	5′-TTACTGGAAAGGCCCAAGCA-3′
Muc5b	5′-CTCTGTACTGCCCCCAGGAT-3′	5′-AAGGTGACATGCCTTGTGGAT-3′
Tgfb1	5′-ACGTGGAAATCAACGGGATCAG-3′	5′-AGTTGGTATCCAGGGCTCTCC-3′
Serpine1	5′-CCGGAATGTGGTCTTCTCTCC-3′	5′-TAGCATCTTGGATCTGCCGC-3′
Col1a1	5′-CACTGCAAGAACAGCGTAGC-3′	5′-AAGTTCCGGTGTGACTCGTG-3′

Supplementary Table 2. Primer sequences used for real-time RT-PCR.

Analysis of infiltrating cells into the lungs

Antibody	Clone	Catalog number	Dilution	Source
Anti-mouse CD16/CD32 antibody	93	101302	1:200	BioLegend
FITC anti-mouse Ly-6G Antibody	1A8	127606	1:500	BioLegend
PerCP/Cyanine5.5 anti-mouse CD11c Antibody	N418	117328	1:200	BioLegend
APC anti-mouse Siglec-F Antibody	REA798	130-112-175	1:200	Miltenyi Biotec
Alexa Fluor 700 anti-mouse CD4 Antibody	GK1.5	100430	1:500	BioLegend
Alexa Fluor 700 anti-mouse I-A/I-E Antibody	M5/114.15.2	107622	1:500	BioLegend
Brilliant Violet 421 anti-mouse I-A/I-E Antibody	M5/114.15.2	107632	1:200	BioLegend
Brilliant Violet 421 anti-mouse CD3 Antibody	17A2	100227	1:200	BioLegend
Brilliant Violet 510 anti-mouse/human CD11b Antibody	M1/70	101263	1:200	BioLegend
Brilliant Violet 605 anti-mouse CD8a Antibody	53-6.7	100744	1:200	BioLegend
PE anti-mouse CD45 Antibody	30-F11	103106	1:200	BioLegend
PE/Cyanine7 anti-mouse CD3 Antibody	17A2	100220	1:200	BioLegend
PE/Cyanine7 anti-mouse CD4 Antibody	RM4-5	100528	1:200	BioLegend

Analysis of T cells infiltrating the lungs

Antibody	Clone	Catalog number	Dilution	Source
**Cell surface antigen staining**				
Anti-mouse CD16/CD32 antibody	93	101302	1:200	BioLegend
APC anti-mouse CD3ε antibody	145-2C11	100312	1:200	BioLegend
Alexa Fluor 700 anti-mouse CD4 antibody	GK1.5	100430	1:500	BioLegend
Brilliant Violet 510 anti-mouse/human CD44 Antibody	IM7	103044	1:200	BioLegend
**Intracellular cytokine staining**				
Brilliant Violet 421 anti-mouse IL-4 Antibody	11B11	504120	1:200	BioLegend
Brilliant Violet 605 anti-mouse IFN-γ Antibody	XMG1.2	505840	1:200	BioLegend
PE anti-mouse/human IL-5 Antibody	TRFK5	504304	1:200	BioLegend
PE-Cyanine7 anti-mouse/human IL-13 Antibody	eBio13A	25-7133-82	1:200	Thermo Fisher Scientific

Supplementary Table 3. Antibodies used for flow cytometry
